# Owner reported prevalence of autonomic signs in epileptic dogs

**DOI:** 10.3389/fvets.2026.1891856

**Published:** 2026-09-03

**Authors:** Christopher Skinner, Michał Czopowicz, Rodrigo Gutierrez-Quintana, Catherine Stalin

**Affiliations:** 1 Small Animal Hospital, School of Biodiversity, One Health and Veterinary Medicine, University of Glasgow, Glasgow, United Kingdom; 2 IVC Evidensia Small Animal Hospital Södra, Stockholm, Sweden; 3 Division of Veterinary Epidemiology and Economics, Institute of Veterinary Medicine, Warsaw University of Life Sciences-SGGW, Warsaw, Poland

**Keywords:** autonomic signs, canine, dog, epilepsy, survey

## Abstract

**Introduction:**

Epilepsy is a common neurological disorder in dogs. Autonomic manifestations are commonly recognised but poorly reported in veterinary literature. This study aimed to determine owner reported prevalence and course of autonomic signs in epileptic dogs.

**Methods:**

A survey for owners of dogs with epilepsy created with Qualtrics was circulated on Facebook between March 2025 and August 2025.

**Results:**

There were 1,154 responses, with 704 included in statistical analysis. The overall frequency of autonomic signs was 697/704 dogs (99.0%). When analysed by body system, gastrointestinal signs [96.9% (682/704)], and urogenital signs [91.1% (641/704)] were most common. Individually, commonly reported signs included ptyalism [87.6% (617/704)], urination [79.1% (556/703)], polydipsia [78.4% (551/703)], mydriasis [71.8% (505/703)], and owner-reported appetite changes [73.9% (520/704)]; the latter could not be confidently attributed to seizure-associated autonomic dysfunction. The least prevalent were priapism [14.6% (66/452)], apnoea [16.7% (117/701)], anisocoria [18.8% (132/702)], cyanosis [20.5% (144/702)], and abdominal pain [21.3% (150/703)]. Antiseizure drugs, defined as any medications used for epileptic seizure control for both acute and maintenance management, were significantly associated with increased prevalence of several autonomic signs. Additionally, mean course of progression (deterioration) rate (3.6%) was significantly higher than mean course of regression (improvement) rate (2.9%; *p* < 0.001). When analysed by body system, overall regression was most common for gastrointestinal [11.2% (79/704)] and urogenital [8.8% (62/704)] autonomic signs, and overall progression was most common for gastrointestinal [15.9% (112/704)] and cardiorespiratory [15.8% (111/704)] autonomic signs.

**Conclusion:**

This study demonstrated that autonomic signs are ubiquitous in epileptic dogs, progression of autonomic signs is uncommon, and progression occurs more often than regression. The prevalence of autonomic signs in epileptic dogs appears to be significantly higher than previously reported. Additionally, changes in the course of autonomic signs indicate the need to monitor autonomic activity in individuals managed for epilepsy, due to the potential impact on quality of life (QoL).

## 1 Introduction

Epilepsy, defined as a disease of the brain characterized by an enduring predisposition to generate epileptic seizures, is one of the most common chronic neurological disorders in dogs (1, 2). Traditionally, the clinical focus in epilepsy management has been the control of motor epileptic seizures and reduction in epileptic seizure frequency (3). However, in both human and veterinary medicine, growing attention is being directed towards the autonomic manifestations of epilepsy, which have important diagnostic, clinical, and QoL implications (1, 4–11). Autonomic changes can occur in both generalised and focal epileptic seizures and reflect interactions between epileptic activity and the central autonomic network (CAN).

Autonomic signs during epileptic seizures include a wide array of clinical signs, triggered by direct activation of the CAN by epileptic activity (7). When epileptic activity engages these regions, the resulting signs can be highly variable, and in humans have been shown to include cardiovascular, respiratory, gastrointestinal, cutaneous, ocular, sexual, and urinary manifestations (7, 12). Dogs presenting with focal epileptic seizures (13) show clinical signs resembling those observed in humans. This overlap may also include other forms of epileptic seizures but has not been well described.

Autonomic manifestations are often readily observed by owners, yet they are rarely systematically recorded or quantified in clinical settings. The presence of these signs has diagnostic value, particularly in differentiating epileptic seizures from other paroxysmal events (e.g., tremors, vestibular signs, syncope, paroxysmal dyskinesias). Furthermore, autonomic signs may precede or outlast the motor components of an epileptic seizure or occur in isolation of motor manifestations (14, 15), providing an opportunity for earlier recognition (10).

Some autonomic signs may be observed interictally and may reflect chronic dysregulation of the autonomic nervous system (10, 14, 16). For example, heart rate variability (HRV) has been used in both human and veterinary epilepsy research to explore these interictal patterns (6). In dogs with idiopathic epilepsy, HRV analysis has shown increased parasympathetic tone, which differs from findings in humans (6). These findings suggest that canine epilepsy may involve species specific patterns of autonomic dysfunction. In humans, there is evidence linking epilepsy with increased risk of sudden unexpected death in epilepsy (SUDEP), a phenomenon believed to involve autonomic dysfunction, particularly cardiorespiratory instability (14). SUDEP includes death related to recurrent unprovoked epileptic seizures, and unexpected death with no obvious identifiable cause that can be found on post-mortem examination (17). In veterinary neurology, an analogous syndrome known as probable sudden unexpected death in epilepsy (pSUDEP) has been proposed and estimated to cause 5–10% of sudden unexpected deaths in epileptic dogs (17, 18).

The implications of autonomic changes in epilepsy extend beyond epileptic seizure classification and pSUDEP risk. These changes can significantly impact QoL for both the dogs and owners (1, 4). Despite this, the impact of autonomic manifestations on QoL has not been formally evaluated in veterinary patients. Studies focusing on epileptic seizure detection (9, 10) and the postictal phase (4) have highlighted the broader QoL implications of epilepsy, yet autonomic signs remain a neglected area.

The objectives of this study were to (i) determine the prevalence of autonomic signs in epileptic dogs as observed by owners; (ii) identify specific characteristics linked with the occurrence of certain groups of autonomic signs; and (iii) determine the course of autonomic signs (progression and/or regression).

## 2 Methods

### 2.1 Survey design & inclusion criteria

A cross-sectional survey for owners of epileptic dogs was created using Qualtrics (Qualtrics, Provo, USA). A required sample size was determined to be at least 385 dogs, assuming an expected prevalence of autonomic signs of 50%, precision of prevalence estimation of ±5%, and a confidence level of 95% (19). Although only approximately 23% of seizuring dogs have shown autonomic signs in a previous small-scale study (13), the minimum required sample size was calculated so that it satisfied statistical conditions even in the most demanding scenario in which 50% of enrolled dogs have a certain autonomic sign. The survey was circulated on multiple Facebook groups for owners of epileptic dogs from March 2025 to August 2025. Participation was voluntary, there were no incentives offered, and responses were anonymised. Each respondent gave consent for their responses to be used as part of the study but could opt out at any time. Providing an answer was not mandatory to continue to the next question. Brief descriptions of generalised, focal and absence epileptic seizures were provided. Inclusion relied on owner-reported diagnoses of epileptic conditions as veterinary records were not evaluated. Responders were included if (i) they gave or had previously given care for a dog diagnosed with recurrent epileptic seizures, and (ii) if they had completed the survey to the satisfaction of Qualtrics which reviews all responses for quality. Ethical approval was granted by the Ethics Committee of the College of Medical, Veterinary and Life Sciences of the University of Glasgow (ethical approval number EA10/25) (Supplementary materials 1, 2).

The survey consisted of 33 questions divided over three parts: part one asked general questions relating to signalment, epileptic seizure onset and description, diagnostic evaluations, and other health conditions; part two enquired about treatments and progression of epileptic seizures; part three queried subgroups of autonomic signs (cardiorespiratory, gastrointestinal, cutaneous, ocular, urogenital) and their course of progression (Supplementary material S1). Tachypnoea was defined as more than 30 breaths per minute, and bradypnoea as less than 10 breaths per minute. For all autonomic signs, owners were asked if signs were observed during or immediately after a seizure. No more specific timeframe or definition was provided for appetite changes, and therefore transient peri−/post-ictal changes could not be reliably distinguished from interictal appetite changes. Questions regarding the frequency of observed signs included six options signs observed always [100% of the time when the owner sees the dog], most of the time [75–99% of the time], often [50–75% of the time], sometimes [25–49% of the time], uncommonly [10–24% of the time], and rarely/never [less than 10% of the time]. Progression questions included three options (better, stayed the same, worse).

### 2.2 Exclusion and classification criteria

Responses were excluded from the study if they were (i) suspected of being created by bots according to Qualtrics™; (ii) provided from the same IP address; (iii) completed within less than 60 s; (iv) below 90% completion rate, i.e., less than 22/24 closed questions in part 1 or less than 44/48 closed questions in part 2. Only 1 entry per an IP address was allowed; otherwise, we only included the response with the highest number of completed questions, or the most recent if the number of completed questions was identical. If the breed was not listed, or a mixture of breeds was reported, they were classified as “Not reported” or “Mixed breed,” respectively. In response to the question regarding epileptic seizure types, respondents were allowed to select all relevant answers. An autonomic sign was only considered present if there was more than 10% prevalence reported.

### 2.3 Statistical analysis

Categorical variables were summarised as frequencies and proportions (%). The 95% confidence intervals (95% CI) for proportions (prevalences) were computed using Wilson’s score method (20). Prevalence of individual autonomic signs, groups of autonomic signs, and change rates were compared first using Cohran’s Q test and, if significant at significance level (α) of 0.05, pairwise comparisons were performed using McNemar’s test at α = 0.05 adjusted by Holm-Bonferroni correction to control for type I statistical error inflation due to multiple comparisons (21). In the analysis of associations between dog characteristics and the occurrence of autonomic signs, first the univariable analysis using the maximum likelihood G test was carried out. The variables, whose *p*-value was below a liberal α of 0.15, were introduced into the multivariable logistic regression analysis to identify a set of variables independently associated with the occurrence of autonomic signs. Both manual forward insertion and manual backward elimination procedures were carried out to identify the optimal multivariable model. Model goodness-of-fit was evaluated using Hosmer-Lemeshow χ^2^ test, Nagelkerke’s pseudo-R^2^ coefficient, and Bayesian information criterion (BIC) (22). The strength of association was expressed as the odds ratio; crude odds ratio (OR) in the univariable analysis and adjusted odds ratio (OR_adj_) in the multivariable analysis. Changes in intensity of autonomic signs were denoted with scores of −1 for regression, +1 for progression, and 0 if they remained unchanged. The overall change in intensity of the entire group of autonomic signs was defined as the sum of scores of all autonomic signs belonging to this group; with the following interpretation: overall regression (improvement) if it was negative and overall progression (deterioration) if it was positive. The change rate (progression, regression rate, and overall change rate) of intensity of autonomic signs was estimated by dividing the number of events (changes, progressions, or regressions) by the total number of valid records regarding changes. The 95% CI for change rates were calculated by dividing appropriate values from the Poisson distribution by the total number of valid records regarding changes (20). Statistical analysis was performed in TIBCO Statistica 13.3 (TIBCO Software Inc., Palo Alto, CA), IBM SPSS Statistics 31 (IBM Corporation, Armonk, NY) and Microsoft Excel.

## 3 Results

### 3.1 Study population selection

**Figure 1:**
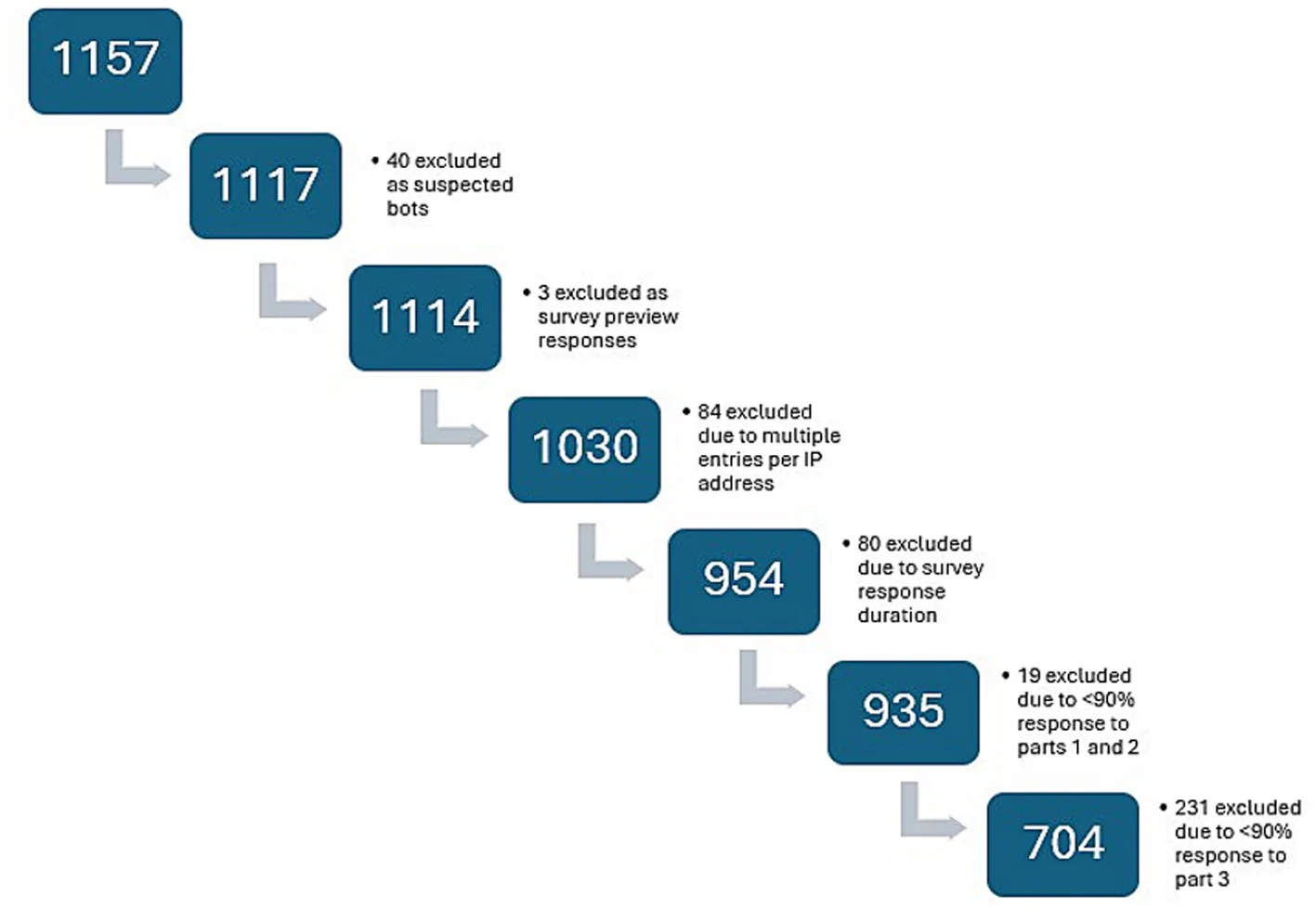
Flow chart showing exclusion of data prior to statistical analysis.

There were 1,157 responses to the survey on Qualtrics and 704 were included in the statistical analysis (Figure 1). Duration of the survey ranged from 3.6 min to 16.8 h with the median of 7.6 min (interquartile range: 6.3 to 9.9 min). Ninety-five per cent of questionnaires were completed within 17 min and 99% within 53 min. The remaining 1% of questionnaires were completed over a longer time than 53 min (Figure 1).

### 3.2 Study population characteristics

Of the reported population, 452/704 dogs (64.2%) were males [352/452 (71.9%) neutered] and 252/704 dogs (35.8%) were females [205/252 (81.4%) spayed]. Most dogs were between 1–6 years old [418/703 (59.5%)], followed by dogs aged 7–10 years [202/703 (28.7%)], more than 10 years [73/703 (10.4%)], and less than 1 year old [10/703 (1.4%)]. Age was not reported for 1 dog. There were 102 breeds reported, including 533/704 (75.7%) pure breeds, 124/704 (17.6%) mixed breeds, and 47/704 (6.7%) unknown. Of 657 known breeds, 299 dogs (45.5%) were classified as large (20–40 kg), 172 (26.2%) as medium (10–20 kg), 106 (16.1%) as small (5–10 kg), 44 (6.7%) as giant (>40 kg), and 36 (5.5%) as toy (<5 kg) (Supplementary Tables S1, S2).

### 3.3 Clinical characteristics

In most dogs [501/704 (71.2%)] epileptic seizures were first observed between 1–6 years old (Supplementary Table S2). Most dogs experienced generalized tonic–clonic epileptic seizures, either alone [427/704 (60.7%)], with focal epileptic seizures [134/704 (19.0%)], or with both focal and absence epileptic seizures [64/704 (9.1%)]. Epileptic seizure frequency during the preceding 6 months was less than 1 seizure per month in 242/703 dogs (34.4%), 1–2 seizures per month in 220/703 (31.3%), 3–4 seizures per month in 158/703 (22.5%), and more than 5 seizures per month in 83/703 (11.8%). Idiopathic epilepsy was the most reported diagnosis [613/704 (87.1%)]. Routine bloodwork was performed in 677/703 dogs (96.3%), while MRI and cerebrospinal fluid analysis were performed in 159/703 (23.0%) and 110/703 dogs (15.7%), respectively. The most used antiseizure medications were phenobarbital [508/704 (72.2%)] and levetiracetam [435/704 (61.8%)]. Concurrent health conditions were reported in 189/704 dogs (26.8%), most commonly dermatologic and gastrointestinal conditions [52/704 (7.4%) each], musculoskeletal conditions [35/704 (5.0%)], and cardiac conditions [33/704 (4.7%)] (Supplementary Table S2).

### 3.4 Prevalence of autonomic signs

**Figure 2:**
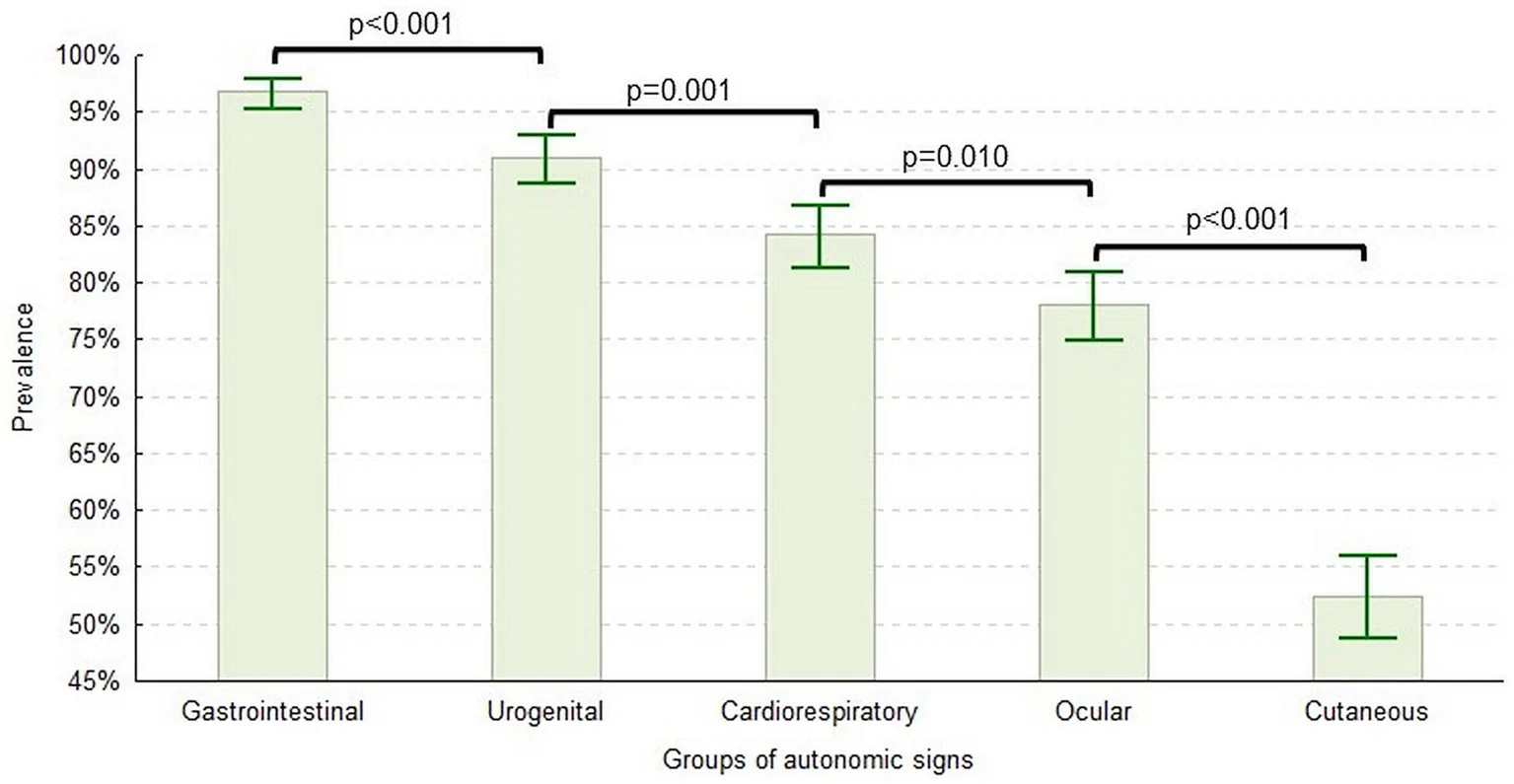
Prevalence of groups of autonomic signs in the study population. Whiskers indicate 95% confidence intervals. The *p*-values come from the McNemar’s test with Holm-Bonferroni correction.

The overall prevalence of at least one autonomic sign was 697/704 dogs [99.0% (95% CI: 98.0, 99.5%)]. When evaluated by body system there was greater variation (Supplementary Table S3). Gastrointestinal signs were seen in 682/704 dogs [96.9% (95% CI: 95.3, 97.9%)], followed by urogenital signs in 641/704 dogs [91.1% (95% CI: 88.7, 92.9%)], cardiorespiratory signs in 593/704 dogs [84.2% (95% CI: 81.4, 86.7%)], ocular signs in 550/704 dogs [78.1% (95% CI: 74.0, 81.0%)], and finally cutaneous signs in 369/704 dogs [52.4% (95% CI: 48.7, 56.1%)] (Figure 2). Typically, respondents reported the occurrence of 2 to 4 individual gastrointestinal signs, 2 urogenital signs, and 1 to 2 autonomic signs from the remaining groups (Supplementary Table S3).

**Figure 3:**
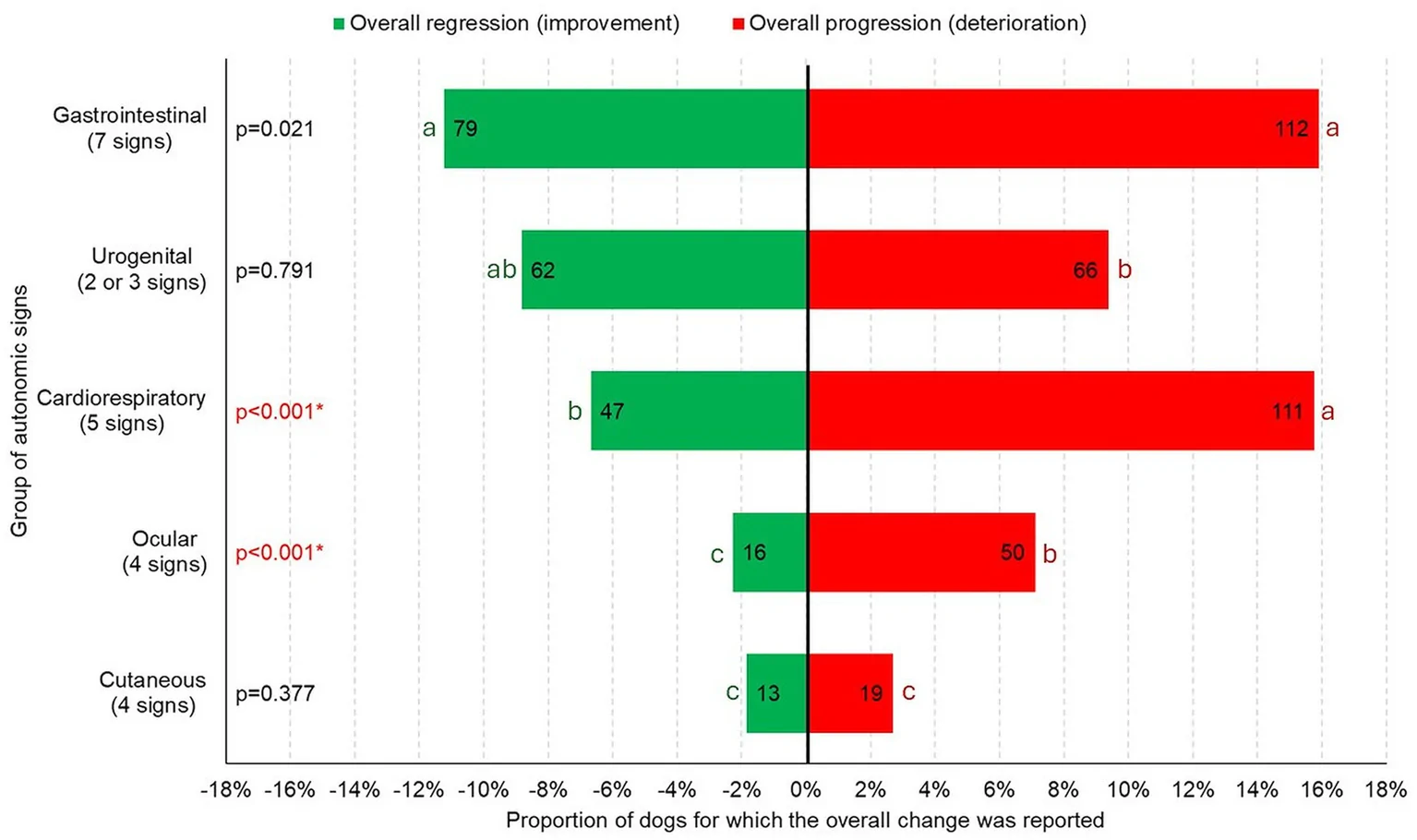
Intensity of autonomic signs observed in the study population.

Individually, the most common signs included ptyalism in 617/704 dogs (87.6%), urination in 556/703 dogs (79.1%), polydipsia in 551/703 dogs (78.4%), and mydriasis in 505/703 dogs (71.8%). Owner-reported appetite changes were present in 520/704 dogs (73.9%); however, these could not be specifically attributed to seizure-associated autonomic dysfunction. The above signs were observed always (100%) or most of the time (75–99%) by at least half of the owners who observed them. The least prevalent were priapism in 66/452 (14.6%), apnoea in 117/701 (16.7%), anisocoria in 132/704 (18.8%), cyanosis in 144/702 (20.5%), and abdominal pain in 150/703 dogs (21.3%). The least prevalent signs were observed uncommonly (10–24%) or rarely/never (<10%) by most owners who observed them (Supplementary Table S4; Figure 3).

### 3.5 Associations between dog characteristics and the occurrence of autonomic signs

In the univariable analysis, the prevalence of gastrointestinal signs was significantly higher in dogs that had GTCS [OR 4.4 (95% CI: 1.5, 12.4); *p* = 0.015], more than one epileptic seizure per month [OR 2.9 (95% CI: 1.2, 6.8); *p* = 0.017], and were treated with antiseizure drugs [OR 4.8 (95% CI: 1.8, 12.9); *p* = 0.005]. Namely phenobarbital [OR 2.7 (95% CI: 1.2, 6.3); *p* = 0.026] and levetiracetam [OR 2.9 (95% CI: 1.2, 7.1); *p* = 0.014] were significantly linked with the occurrence of gastrointestinal autonomic signs (Supplementary Table S5). In the multivariable analysis, two of these factors proved to be independently and positively associated with the occurrence of gastrointestinal signs: occurrence of GTCS [OR_adj_ 3.2 (95% CI: 1.1, 9.6); *p* = 0.040], and treatment with any antiseizure drugs [OR_adj_ 3.8 (95% CI: 1.4, 10.7); *p* = 0.011] (Supplementary Table S6). At the level of individual gastrointestinal autonomic signs, the occurrence of GTCS was significantly associated with higher prevalence of ptyalism [OR 2.9 (95% CI: 1.5, 5.8); *p* = 0.004] and defecation [OR 6.4 (95% CI: 3.0, 13.3); *p* < 0.001], while the use of any antiseizure drugs was significantly associated with higher prevalence of ptyalism [OR 2.1 (95% CI: 1.1, 4.2); *p* = 0.040], appetite changes [OR 2.8 (95% CI: 1.6, 4.9); *p* < 0.001], and flatulence [OR 2.1 (95% CI: 1.1, 3.9); *p* = 0.018].

The prevalence of urogenital signs found in univariable analysis was significantly higher in entire males [OR 7.4 (95% CI: 1.8, 30.6); *p* < 0.001], dogs with GTCS [OR 4.0 (5% CI: 1.9, 8.1); *p* = 0.001], and dogs treated with phenobarbital [OR 2.0 (95% CI: 1.1, 3.3); *p* = 0.016], but significantly lower in dogs aged over 10 years old [OR 0.4 (95% CI: 0.2, 0.8); *p* = 0.011] (Supplementary Table S7). Multivariable analysis showed that three of these factors proved to be independently associated with the occurrence of urogenital autonomic signs; entire male status [OR_adj_ 6.2 (95% CI: 1.5, 26.0); *p* = 0.012] and GTCS [OR_adj_ 3.5 (95% CI: 1.7, 7.2); *p* = 0.001] were positively associated, while age over 10 years was negatively associated [OR_adj_ 0.4 (95% CI: 0.2, 0.8); *p* = 0.011] (Supplementary Table S8). At the level of individual urogenital autonomic signs, the occurrence of GTCS was significantly associated with higher prevalence of urination [OR 4.8 (95% CI: 2.6, 8.7); *p* < 0.001] and polydipsia [OR 3.1 (95% CI: 1.7, 5.7); *p* < 0.001], while both entire male status and age over 10 years old were significantly associated only with urination [OR 2.2 (95% CI: 1.3, 3.9); *p* = 0.003] and [OR 0.5 (95% CI: 0.3, 0.8); *p* = 0.005, respectively].

In regard to cardiorespiratory autonomic signs, the univariable analysis revealed that the prevalence of cardiorespiratory autonomic signs was significantly higher in medium, large and giant breed dogs [OR 2.0 (95% CI: 1.3, 3.2); *p* = 0.011], especially in Labrador Retrievers [OR 4.1 (95% CI: 1.0, 17.0; *p* = 0.019)]. Prevalence was also increased in dogs with GTCS [OR 3.0 (95% CI: 1.6, 5.6); *p* = 0.002], dogs experiencing more than one episode per month [OR 1.7 (95% CI: 1.2, 2.6); *p* = 0.009], and dogs treated with any antiseizure drugs [OR 2.4 (95% CI: 1.3, 4.5); *p* = 0.009], in particular levetiracetam [OR 1.7 (95% CI: 1.2, 2.6); *p* = 0.008], benzodiazepines [OR 1.9 (95% CI: 1.1, 3.4); *p* = 0.018], and zonisamide [OR 2.4 (95% CI: 1.1, 5.4); *p* = 0.016] (Supplementary Table S9). With multivariable analysis the occurrence of cardiorespiratory autonomic signs proved to be significantly independently and positively associated with GTCS [OR_adj_ 2.6 (95% CI: 1.4, 5.1); *p* = 0.003], epileptic seizure frequency of one or more episodes per month [OR_adj_ 1.6 (95% CI: 1.1, 2.5); *p* = 0.028], treatment with levetiracetam [OR_adj_ 1.6 (95% CI: 1.0, 2.4); *p* = 0.041], and Labrador Retriever breed [OR_adj_ 4.5 (95% CI: 1.1, 19.0); *p* = 0.042] (Supplementary Table S10). At the level of individual cardiorespiratory autonomic signs, all the characteristics identified as significantly associated were in fact linked to higher prevalence of tachypnoea; GCTS had an OR of 2.8 (95% CI: 1.6, 5.1; *p* = 0.001), epileptic seizure frequency of one or more episodes per month had an OR of 2.4 (95% CI: 1.7, 3.4; *p* < 0.001), treatment with levetiracetam had an OR of 1.6 (95% CI: 1.1, 2.2; *p* = 0.007), and Labrador retriever breed had an OR of 2.5 (95% CI: 1.1, 5.6; *p* = 0.020).

The prevalence of ocular autonomic signs upon performing univariable analysis was significantly higher in dogs having more than five episodes per month [OR 2.2 (95% CI: 1.1, 4.4); *p* = 0.015], and in dogs treated with benzodiazepines [OR 1.8 (95% CI: 1.1, 2.8); *p* = 0.019] and zonisamide [OR 2.0 (95% CI: 1.0, 3.7); *p* = 0.027] (Supplementary Table S11). Multivariable analysis found that the occurrence of ocular autonomic signs proved to be significantly independently and positively associated with having more than five episodes per month [OR_adj_ 2.1 (95% CI: 1.1, 4.2); *p* = 0.033] and treatment with benzodiazepines [OR_adj_ 1.7 (95% CI: 1.1, 2.7); *p* = 0.036] (Supplementary Table S12). At the level of individual ocular autonomic signs, both the occurrence of more than five episodes per month and treatment with benzodiazepines were significantly associated with higher prevalence of miosis [OR 1.8 (95% CI: 1.2, 2.9); *p* = 0.013 and OR 1.6 (95% CI: 1.1, 2.3); *p* = 0.025, respectively] and lacrimation [OR 2.5 (95% CI: 1.6, 4.0); *p* < 0.001 and OR 2.0 (95% CI: 1.4, 2.8); *p* < 0.001, respectively].

After carrying out univariable analysis, the prevalence of cutaneous autonomic signs was significantly higher in medium, large, and giant dogs [OR 1.8 (95% CI: 1.2, 2.6); *p* = 0.003], and in dogs with other reported dermatologic conditions [OR 1.8 (95% CI: 1.0, 3.2); *p* = 0.050] (Supplementary Table S13). Both these factors were confirmed to be independently associated with the occurrence of cutaneous autonomic signs in the multivariable analysis; [OR_adj_ 1.9 (95% CI: 1.3, 2.7); *p* = 0.002] and [OR_adj_ 1.9 (95% CI: 1.1, 3.5); *p* = 0.032], respectively (Supplementary Table S14). At the level of individual cutaneous autonomic signs, only the medium, large, and giant body size was significantly associated with higher prevalence of piloerection [OR 1.7 (95% CI: 1.1, 2.6); *p* = 0.013]. Presence of other dermatologic conditions was not significantly associated with any individual autonomic sign from this group.

### 3.6 Course of autonomic signs

#### 3.6.1 Individual autonomic signs

For the total number of 16,630 valid records regarding changes (potential change of 23 autonomic signs in 701 to 704 dogs and 1 autonomic sign [priapism] in 451 males), 1,096 changes (490 regressions and 606 progressions) were recorded. This amounted to a mean change rate of 6.6% (95% CI: 6.2, 7.0%) with a mean of 1.56 (95% CI: 1.47, 1.65) changes per dog. Progression (deterioration) was significantly more common (mean progression rate of 3.6% [95% CI: 3.4, 4.0%] with a mean of 0.86 [95% CI: 0.79, 0.93] progressions per dog) than regression (improvement) (mean regression rate of 2.9% [95% CI: 2.7, 3.2%] with a mean of 0.70 [95% CI: 0.64, 0.76] regressions per dog; *p* < 0.001).

**Figure 4:**
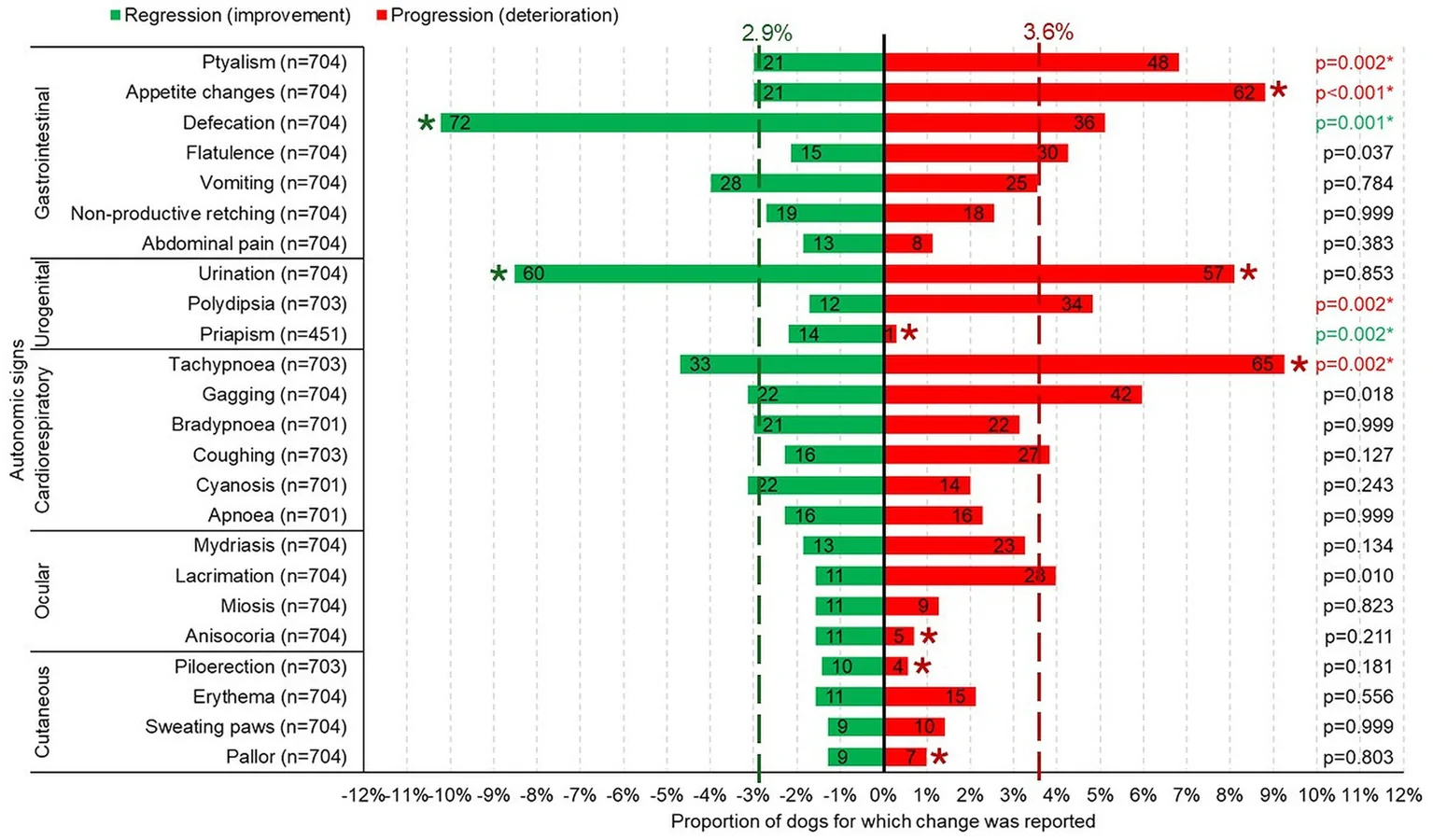
Proportion of dogs for which regression (improvement) and progression (deterioration) of autonomic signs were reported. Absolute numbers of dogs are presented on the bars. The *p*-values come from McNemar’s test and refer to the comparison of proportions of dogs with regression and progression. The color *p*-values are significant at *α* = 0.05 modified by Holm-Bonferroni correction for multiple comparisons – Green color indicates significantly higher proportion of regressions (improvements) while red color indicates significantly higher proportion of progressions (deteriorations). Dashed vertical lines indicate the mean regressions rate (green) and mean progressions rate (red) in the study population. Asterisks indicate autonomic signs for which proportion of regressions (green) or progressions (red) were significantly different from the respective mean rate (*α* = 0.05 modified by Holm-Bonferroni correction for multiple comparisons).

Regression was reported significantly more often than progression in only two individual autonomic signs, defecation (*p* = 0.001), and priapism (*p* = 0.002). Defecation and urination were the two autonomic signs whose regression was significantly more common than the mean regression rate; they were reported to regress in 10.2% of dogs (72/704) and 8.5% of dogs (60/704), respectively (Figure 4).

Progression was reported significantly more commonly than regression for ptyalism (*p* = 0.002), owner-reported appetite changes (*p* < 0.001), polydipsia (*p* = 0.002), and tachypnoea (*p* = 0.002). Progression was reported significantly more often than the mean progression rate for tachypnoea [change reported in 9.2% of dogs (65/703); *p* < 0.001], owner-reported appetite changes [8.8% (62/704); *p* < 0.001], and urination [8.1% (57/704); *p* < 0.001]. Conversely, progression of priapism [0.2% (1/451); *p* < 0.001], piloerection [0.6% (4/703); *p* < 0.001], anisocoria [0.7% (5/704); *p* = 0.001], and pallor [1.0% (7/704); *p* = 0.002] was significantly less common than the mean progression rate (Figure 4).

#### 3.6.2 Group level

**Figure 5:**
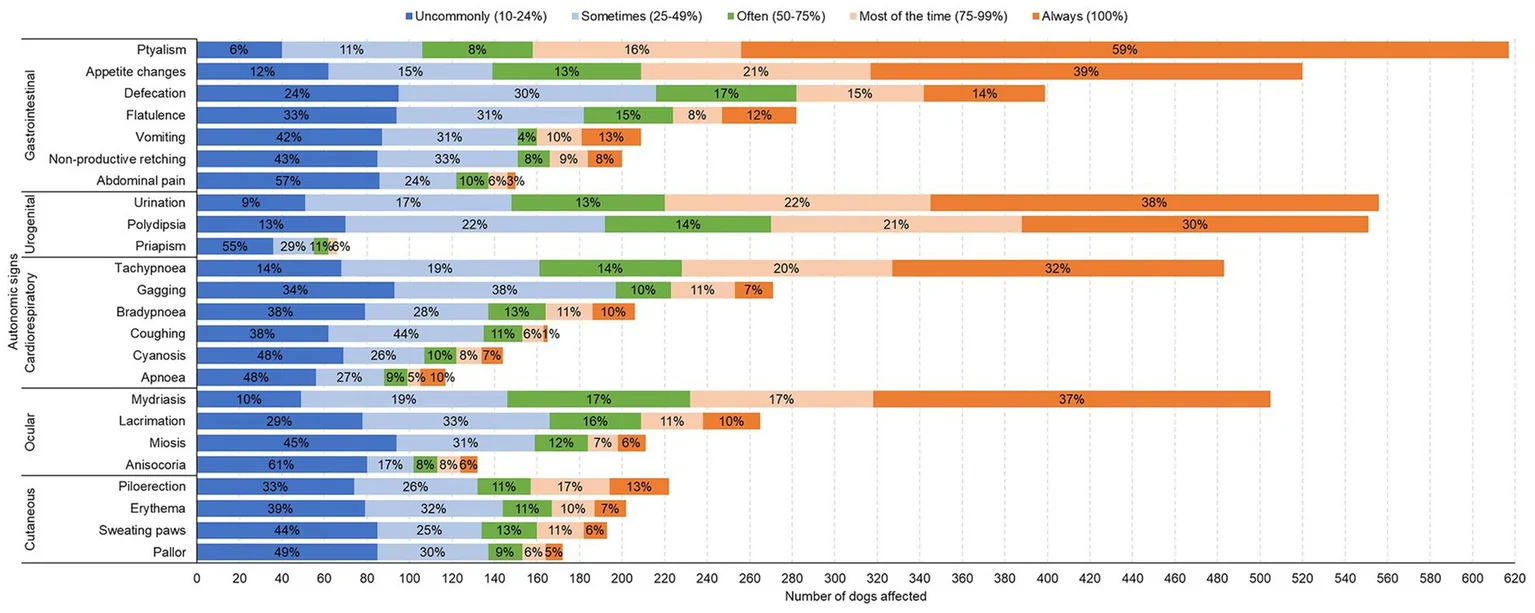
Proportion of dogs for which overall regression (improvement) and progression (deterioration) of groups of autonomic signs were reported. Absolute numbers of dogs presented on the bars. The *p*-values come from McNemar’s test and refer to the comparison of proportions of dogs with overall regression and overall progression; the color *p*-values are significant at *α* = 0.05 modified by Holm-Bonferroni correction for multiple comparisons; red color indicates significantly higher proportion of overall progressions. Small letters refer to between-group comparisons within overall regressions (green letters) and within overall progressions (red letters); groups marked by different letters differed significantly (McNemar’s test at *α* = 0.05 modified by Holm-Bonferroni correction for multiple comparisons).

The overall change rate was highest in the gastrointestinal group [27.1% of dogs (191/704) with overall change], followed by cardiorespiratory [22.4% (158/704)] and urogenital [18.2% (128/704)] (Figure 5). The overall change rate was significantly lower in ocular [9.4% (66/704); *p* < 0.001] and cutaneous groups (4.5%; 32/704; *p* = 0.001). Overall progression was significantly more common than overall regression for cardiorespiratory (*p* < 0.001) and ocular groups (*p* < 0.001). Overall regression was most common in the gastrointestinal group [11.2% (79/704)], followed by urogenital [8.8% (62/704)], cardiorespiratory [6.7% (47/704)], ocular [2.3% (16/704)], and cutaneous [1.8% (13/704)] groups. The overall regression rate was significantly higher in the former three groups than in the latter two (*p* < 0.001). Overall progression was most common in the gastrointestinal [15.9% (112/704)] and cardiorespiratory groups [15.8% (111/704)], followed by significantly lower overall progression rate in urogenital [9.4% (66/704)] and ocular groups [7.1% (50/704); *p* < 0.001] and the lowest in the cutaneous group [2.7% (19/704); *p* < 0.001].

## 4 Discussion

The present study shows that autonomic signs are observed by virtually all owners of epileptic dogs. Gastrointestinal and urogenital signs were particularly common. Few owners noticed changes in these signs over time, but when changes occurred, worsening was reported more often than improvement. Defecation and urination were the signs that most frequently regressed, while urination, tachypnoea, ptyalism and gagging commonly showed progression. Owner-reported appetite changes were also common and showed progression; however, these findings cannot be confidently attributed to seizure-associated autonomic dysfunction because effects of antiseizure medication and interictal appetite changes cannot be excluded. A previous study reported a frequency of 16/70 (23%) of client-owned dogs displayed autonomic signs but did not explore their duration, severity, or impact on the owner’s perception of QoL (13). The findings of this study have improved our understanding of the prevalence of autonomic activity in dogs with epilepsy.

Findings from our data illustrate a wide variation in prevalence of autonomic signs, both individually and by group. To the author’s knowledge, frequency of autonomic signs has not been well documented in human literature, despite multiple publications characterising the autonomic signatures of epileptic patients (7, 23). In addition to their prevalence, we identified several associations (Table 1). Gastrointestinal signs were significantly positively associated with GTCS, having more than one epileptic seizure per month, and the use of antiseizure drugs (namely phenobarbital and levetiracetam). This could be due to multiple factors, including increased involvement of the CAN during generalised epileptic seizure activity compared to non-generalised epileptic seizures, and potentially due to the side effects of antiseizure drugs (e.g., it is well recognised that phenobarbital may cause polyphagia) (24). The impact of antiseizure drugs in epileptic humans is debated, but it is generally theorised that they influence autonomic dysregulation (25). Urogenital signs were significantly more prevalent in male entire dogs, dogs under 10 years of age, dogs with GTCS, and dogs on phenobarbital. A postulated reason for the association with entire male dogs may be the presence of higher circulating testosterone concentrations, thereby increasing urinary bladder muscle tone, which may lead to increased activation of urinary bladder muscles via autonomic pathways in entire male dogs. Older dogs may have age-related degeneration of urinary bladder muscles, declining sex hormones, and degeneration of nerves innervating the bladder, possibly leading to reduced ability to produce urogenital signs in response to epileptic seizure activity and dysregulated autonomic pathways compared to younger dogs. GTCS may cause more widespread activation of the cerebral cortex, which may increase the chance of triggering pathways that produce urogenital signs compared to focal or absence epileptic seizures. The use of phenobarbital, and perhaps other antiseizure drugs, may also influence urogenital signs independently of epileptic seizure activity (e.g., polyuria secondary to polydipsia, which is a known side effect of phenobarbital) (24). Concurrent urogenital disease may also influence the prevalence of urogenital signs. Cardiorespiratory signs were significantly positively associated with body weight over 10 kg, Labrador Retrievers, presence of GTCS, presence of more than one epileptic seizure per month, and the use of antiseizure drugs (particularly zonisamide and levetiracetam). The link between increased body size of any breed, the Labrador Retriever breed, and cardiorespiratory signs, may be due to increased oxygen demand due to greater total muscle mass, physiological differences in heart and lung function (e.g., lower heart rate and higher stroke volume in larger dogs), and increased body volume to surface area inhibiting heat loss and resulting in increased cardiorespiratory signs. Other large breed dogs (i.e., over 10 kg body weight) may also have increased prevalence of cardiorespiratory signs, however no other breeds were specifically found to have a statistically significant association. Labrador Retrievers may have been overrepresented due to factors such as increased body condition score, underlying cardiorespiratory diseases, or this may have been due to chance. Body condition score was not evaluated in this study, therefore any difference from other breeds is merely speculation. The presence of an association between cardiorespiratory signs and more than one epileptic seizure per month may be linked to increased use of antiseizure drugs, such as respiratory depression with the use of benzodiazepines (26). Consideration should also be given to concurrent cardiorespiratory disease that is unrelated to epileptic seizures. Ocular signs were significantly associated with having more than five epileptic seizures per month, and with the use of benzodiazepines and zonisamide. As above, these links could be related to antiseizure drugs, possibly due to increased doses of these drugs with increased epileptic seizure frequency, although dosing was not evaluated in this study. For example, it is possible that there is increased mydriasis secondary to diazepam use, although to the author’s knowledge this has not been studied in epileptic dogs. It is noted in human literature that benzodiazepines may have a central parasympatholytic effect (27). This may theoretically lead to mydriasis; however, it is likely a multifactorial relationship and is incompletely understood. Cutaneous signs were significantly associated with a body weight greater than 10 kg and a history of dermatological disease. It was theorised that increased body size would be linked to decreased ability to dissipate heat, increased skin thickness, and possibly observer bias due to increased visibility of these signs in larger dogs. A history of dermatological disease was thought to be linked due to a combination of sensitisation of affected areas and secondary changes (e.g., increased vascularity, increased presence of immune cells and inflammatory cytokines). It should be considered that any of the described associations may be coincidental, especially given multiple statistical analyses which could result in inflation of type I statistical error. The most common association that was repeatedly identified was the use of antiseizure drugs, suggesting the prevalence of autonomic signs is strongly influenced by the treatments being used to manage epileptic seizures. In the future, if supported by further evidence, autonomic signs may warrant greater consideration when evaluating antiseizure drug therapy in dogs with epileptic seizures. However, based on the current evidence, this should be regarded only as a potential consideration for future clinical management rather than a formal recommendation.

**Table 1:** Summary of significant associations between demographic and clinical characteristics of dogs and the presence of five groups of autonomic signs.

Groups of autonomic signs	Characteristics of dogs							
	GTCS	Insufficiently controlled epilepsy	Antiseizure drug treatment	Entire males	Age >10 years	Labrador retrievers	Medium, large and giant breeds	Skin and ear problems
Gastrointestinal signs	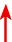		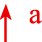					
Urogenital signs	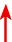			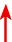	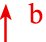			
Cardiorespiratory signs	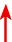	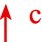	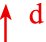			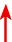		
Ocular signs		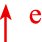	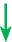					
Cutaneous signs							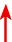	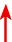

^a^≥1 episode/month; ^b^> 5 episodes/month; ^c^Phenobarbital and levetiracetam most suspected; ^d^levetiracetam and zonisamide most suspected; ^e^benzodiazepines most suspected.

Red indicates an increased association, and green a decreased association.

Our findings demonstrated changes in the course of autonomic signs. There were several differences described in the results that cannot be easily explained with the current understanding of autonomic dysfunction in epileptic dogs. It is possible there are progressively altered associations between structures within the CAN during subsequent epileptic seizures, leading to upregulation or downregulation within these neural circuits. Another possibility to consider is that these changes are due to antiseizure drugs, and perhaps those individuals with progression or regression of epileptic seizure management have had changes to their medication protocol. For example, an increased phenobarbital dose may have caused or worsened polyphagia. In turn, any misunderstanding of the survey questions may lead to inaccurate reporting of appetite changes during or immediately after seizures. This link was not evaluated in this study and could be investigated in future as a prospective study that follows autonomic signs over time alongside seizure management and changes in antiseizure drugs. Additional possibilities include observational biases for more visible autonomic signs, evolution of owner understanding of autonomic signs, patient age, comorbidities, and natural fluctuations of underlying conditions (e.g., idiopathic epilepsy). The course of autonomic signs during epileptic seizures has not been described in veterinary medicine to the author’s knowledge.

Several limitations were noted with this study. There are many potential sources of bias in owner surveys. Reporting of epileptic seizure presence and type relied on owners, based on brief descriptions provided in the survey. As a result, there may have been inclusion of non-epileptic events or unrelated gastrointestinal, cardiorespiratory, urogenital, ocular or dermatological signs, which may have falsely increased the reported prevalence of autonomic signs. There was also no specific time frame defined for autonomic signs, allowing potential misunderstanding about what is considered “during or immediately after having a seizure.” The time frame for autonomic signs was noted as “during or immediately after having a seizure,” therefore there was scope for misinterpretation of what was considered immediate (e.g., 5 min, 30 min, 2 h, etc.). In the human literature, there are well-documented associations between epileptic seizures, mesial temporal lobe epilepsy and autonomic signs, such as autonomic epileptic seizures which is seen with Panayiotopoulos syndrome (14). The manifestation of autonomic epileptic seizures without motor signs may be easily missed and is likely underreported in the veterinary literature, potentially impacting results of this study. The timing of autonomic signs in relation to epileptic seizures (i.e., before, during or after) was not investigated in this study and could therefore influence owner reporting in the survey. There is significant potential for misreporting of medical histories, over and under reporting, recall bias for certain clinical features over others, recall bias for historic features (e.g., from several years prior to completion of the survey), and limited ability of owners to monitor some autonomic signs such as heart rate. A volunteer bias exists, as owners willing to participate may be more engaged, motivated and invested in research. The survey was circulated on Facebook groups for dogs with epilepsy, including all epileptic dogs regardless of underlying condition, hence a source of selection bias was included. Dogs with idiopathic, structural and reactive conditions were not excluded as complete medical records were not available to exclude structural and reactive conditions, and further investigations were not performed on all dogs as reported by owners. Therefore, these cases could not be reliably excluded in a survey study. Dogs with structural and reactive conditions may have experienced autonomic signs related to the underlying process itself, instead of or in addition to epileptic seizure activity, which may in turn influence epileptic seizure type, severity and duration, medication usage (including peri-ictal and maintenance medications), and owner-reported clinical signs. As a result, there are several possible confounding biases. The convenience of completing this survey must be considered, hence the survey length was kept to an estimated length of under ten minutes. There are likely impacts of owner management during and after epileptic seizures that may impact their observations. Additionally, non-motor epileptic seizures are more easily missed, especially absence epileptic seizures and focal autonomic epileptic seizures. Closed questions were almost exclusively used; however, this introduces an element of measurement bias. Questions may be misinterpreted by owners, leading to incorrect and/or incomplete answers. For example, progression of autonomic signs could be incorrectly interpreted as evolution within the peri-ictal period of a single epileptic seizure. Some signs may be difficult for owners to perceive, which may artificially alter our findings (e.g., abdominal pain). Autonomic activity may be affected by anti-seizure drugs, the effects of which cannot be differentiated from dysregulation of autonomic function due to epileptic seizure activity (e.g., polyphagia secondary to diazepam, diarrhoea secondary to levetiracetam, polydipsia secondary to potassium bromide, vomiting secondary to zonisamide, etc.). Benzodiazepines were reported as a group, however there was no further information obtained about protocols for their use. The use of benzodiazepines, in addition to longer term antiseizure drugs, should be considered as potential factors that may influence the reported autonomic activity. Regarding appetite changes, there was potential for misinterpretation of the timeframe of these changes, given they may occur as a consistent interictal sign out with epileptic seizures, as a transient change post-ictally, or in both situations. Consequently, the appetite-change findings cannot be confidently interpreted as evidence of seizure-associated autonomic dysfunction. Dogs may have received multiple antiseizure drugs, which is typically encountered with more frequent and/or prolonged epileptic seizures. Additional factors related to the impact of antiseizure drugs on autonomic signs includes epileptic seizure frequency and duration, the type and number of drugs used, owner compliance, heterogeneity in medication protocols, duration of treatment, serum drugs levels, and how recently changes had been made to medications. This information was not obtained in this survey. The Likert scale used in this survey did not allow respondents to state firmly that their dog never had a certain sign; less than 10% prevalence was considered negligible in this study, therefore those signs that had less than 10% prevalence or those that were never present were combined into the category rarely/never. A counterpoint to the limitations of survey studies is that a much larger cohort was able to be recruited; the relatively large number of reported dogs in this survey may diminish incorrectly reported information. This was an observational study; hence its practical application is limited to advice for owners and awareness for clinicians managing dogs with epileptic seizures. This may be important, as there are many difficulties for owners in managing dogs with epileptic seizures. The clinical focus has historically been on managing motor activity, yet autonomic signs are shown to be significant variables in owner-perceived QoL for their dogs (4). Therefore, changes in the course of autonomic signs may have an impact on QoL. We demonstrated that certain autonomic signs were more prevalent according to owner reporting, and that the course of autonomic signs may change over time. Future studies may be able to elucidate the timing of these signs (i.e., before, during or after epileptic seizures) and their significance, interplay between autonomic signs and anti-seizure medications, where these may originate neuroanatomically, why autonomic signs differ in prevalence, and why some signs show a course of progression and/or regression.

We present data from a survey of client-owned dogs describing the prevalence and course of owner-reported signs potentially associated with autonomic activity in dogs with epileptic seizures. This information has immediate practical use in informing owners of epileptic dogs about potential autonomic signs they may observe and can be useful as a monitoring tool for these patients. The survey study design limits the reliability of this data. This information has not been previously reported and represents an important first step in understanding the nature, neuroanatomical distribution, evolution, risk and management of dysautonomia in dogs with epileptic seizures as an important area of future research. Autonomic signs in epileptic dogs open a unique window into the CAN and may be a useful tool for gaining a better understanding of functional anatomy and pathophysiology.

## Data Availability

The original contributions presented in the study are included in the article/Supplementary material, further inquiries can be directed to the corresponding author/s.
