# Supplementary material for: Owner reported prevalence of autonomic signs in epileptic dogs
Source: Front Vet Sci. 2026 Sep 3;13:1891856. doi: 10.3389/fvets.2026.1891856 (PMC13582489; doi:10.3389/fvets.2026.1891856)

Q1 - I have read and agreed to participate anonymously in this survey.

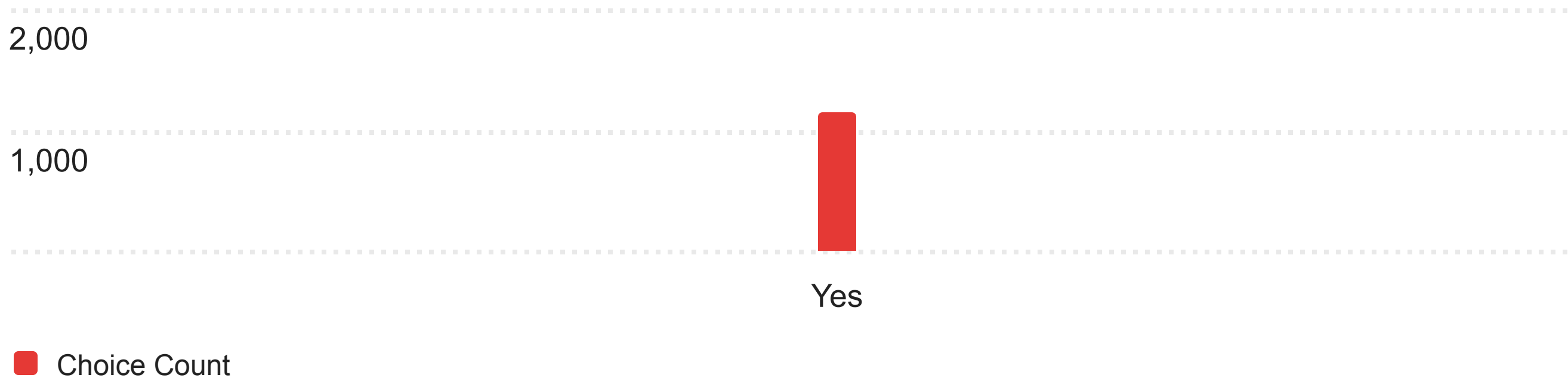

Q2 - How old is your pet?

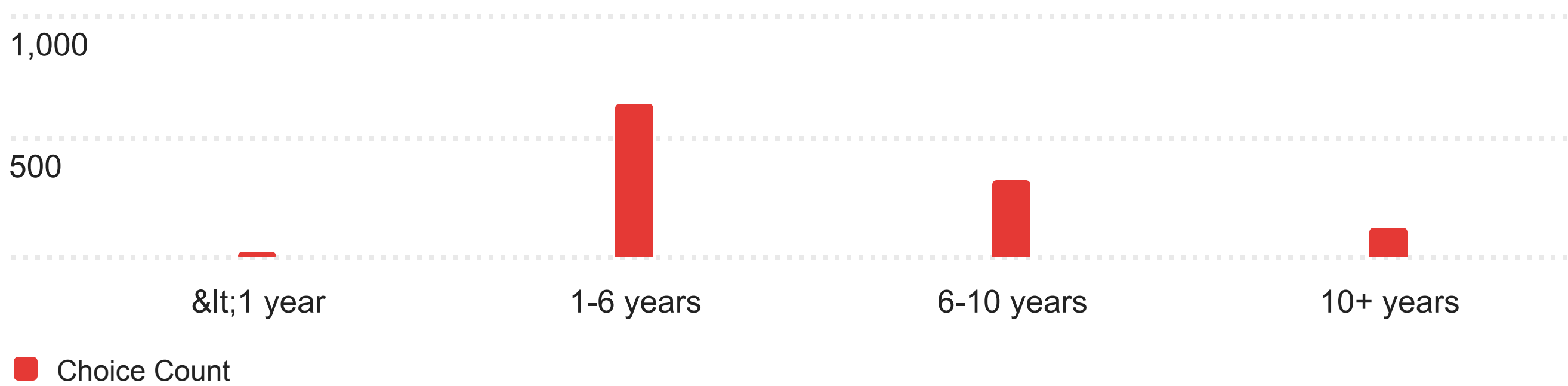

Q3 - What is your pet's gender?

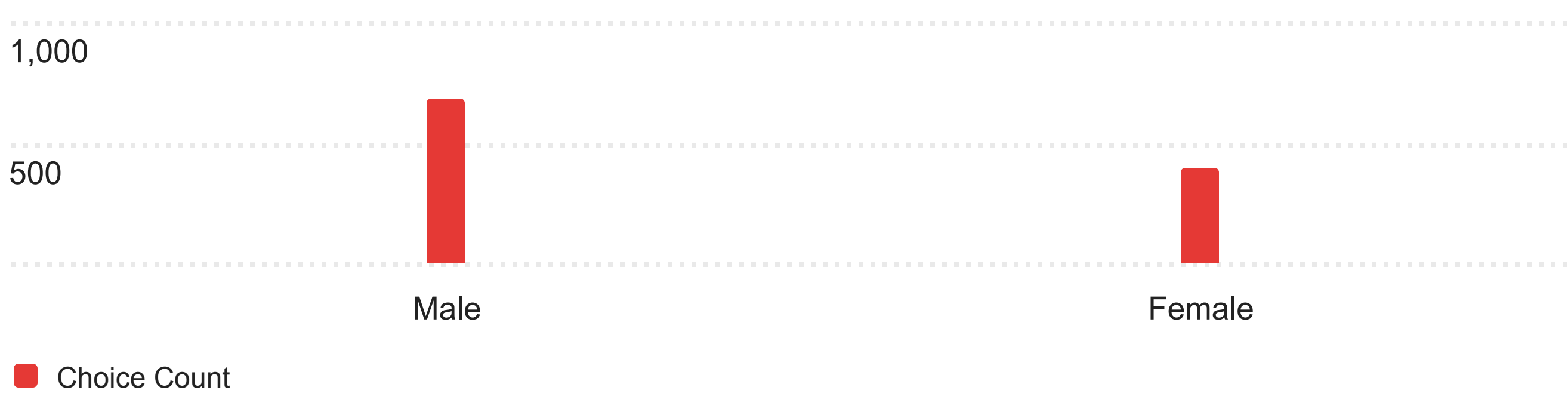

Q5 - Has your pet been spayed/neutered/castrated?

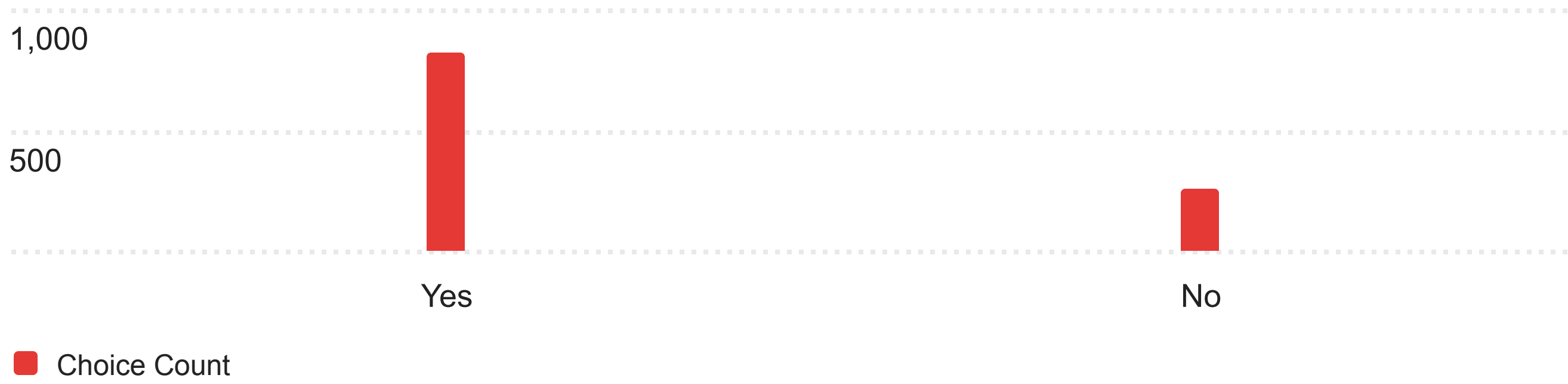

Q6 - What was the approximate age your pet began having seizures?

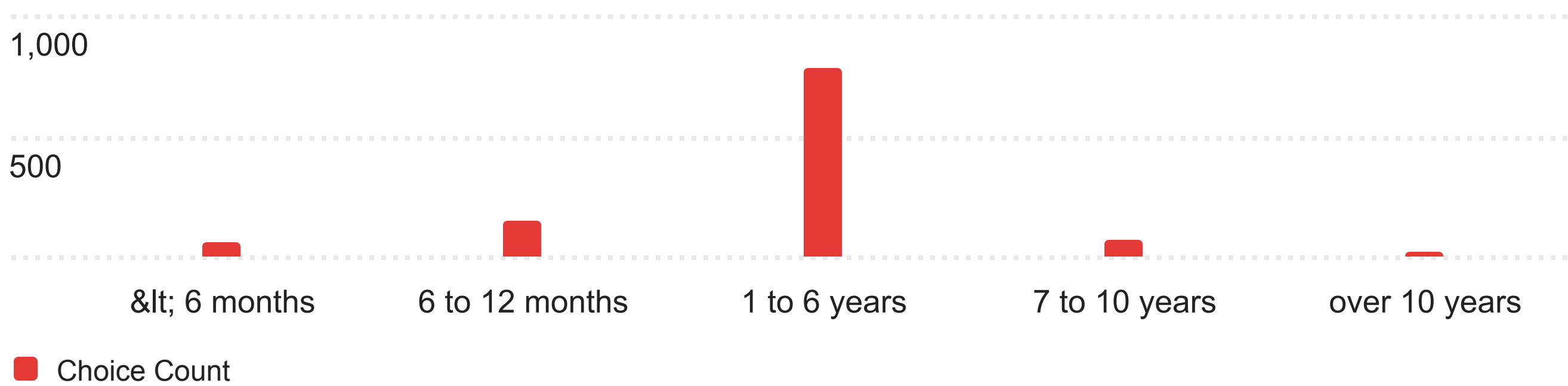

Q7 - What type of seizures does your pet have (please select all relevant answers)?

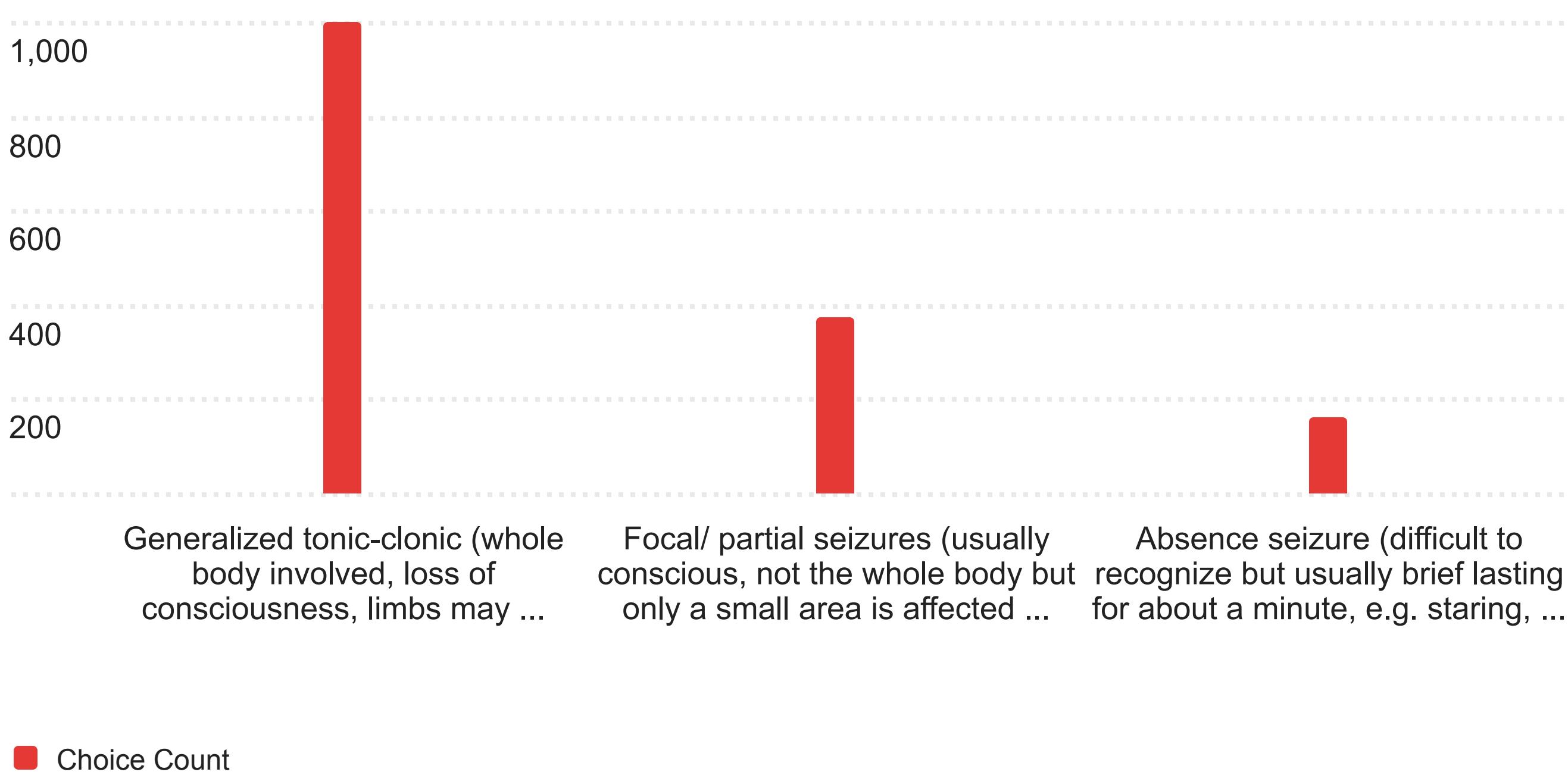

Q8 - Has your pet ever required hospitalisation because of his/her seizures?

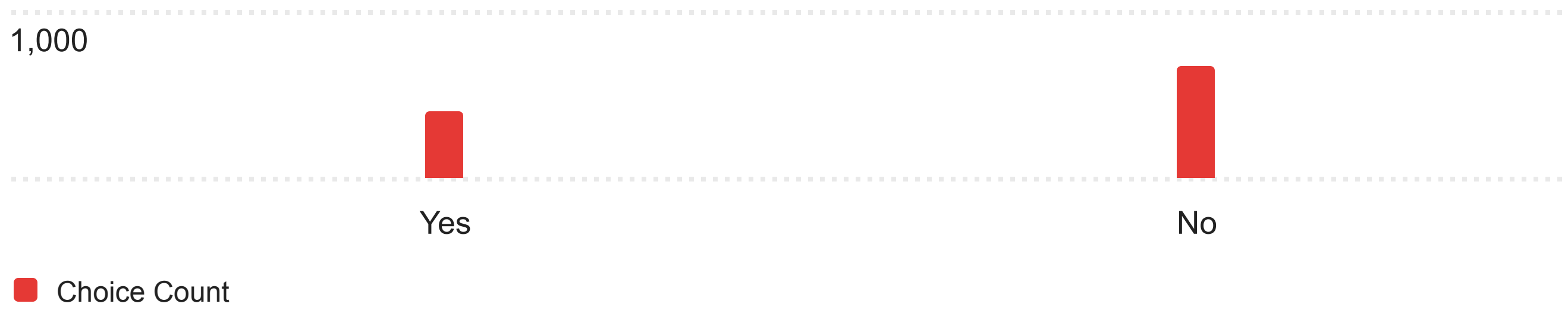

Q9 - Has your pet had a seizure lasting greater than 5 minutes?

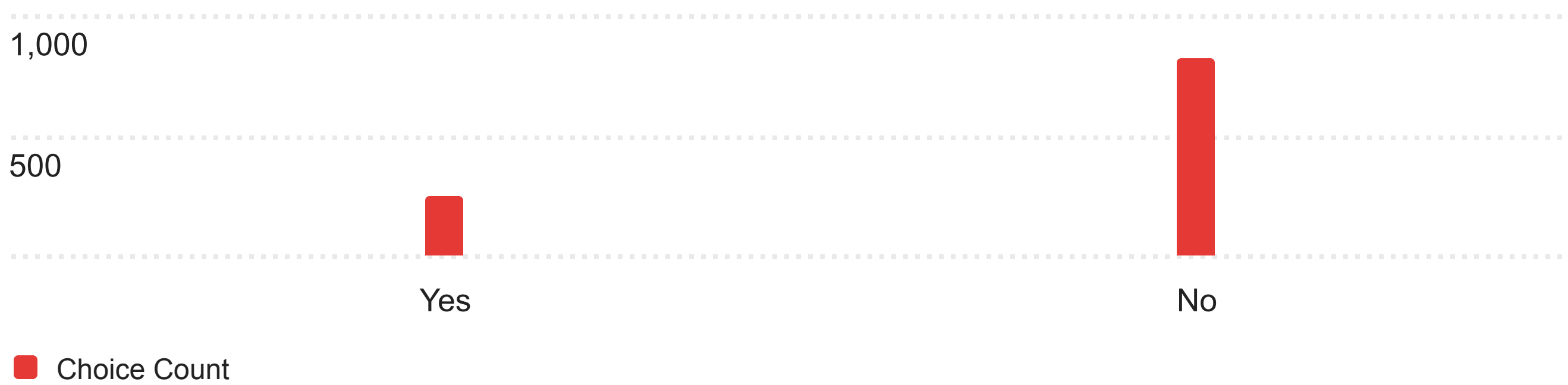

Q10 - What is the longest a seizure has gone on for?

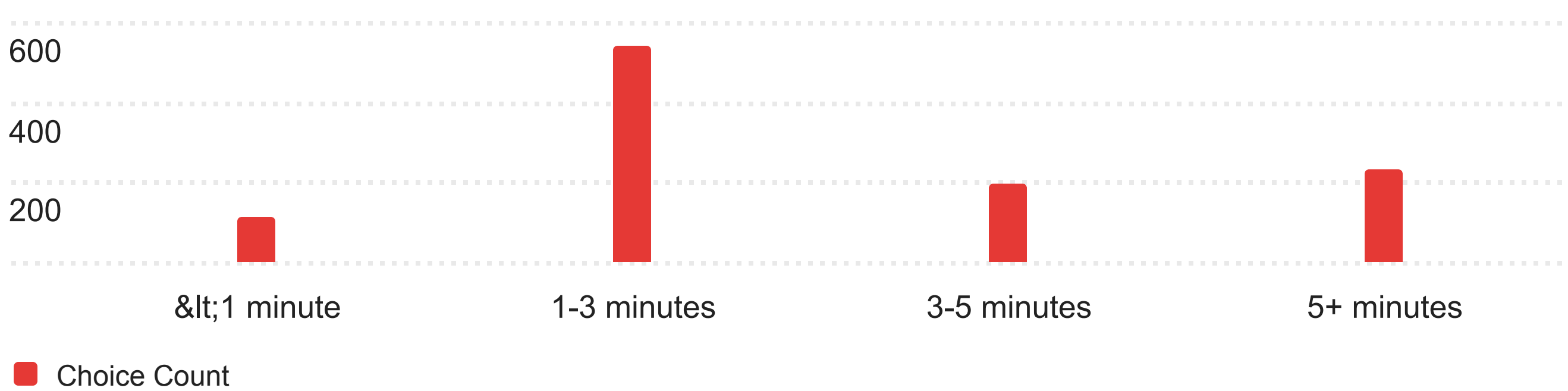

Q11 - Has your pet had two or more seizures within 24 hours?

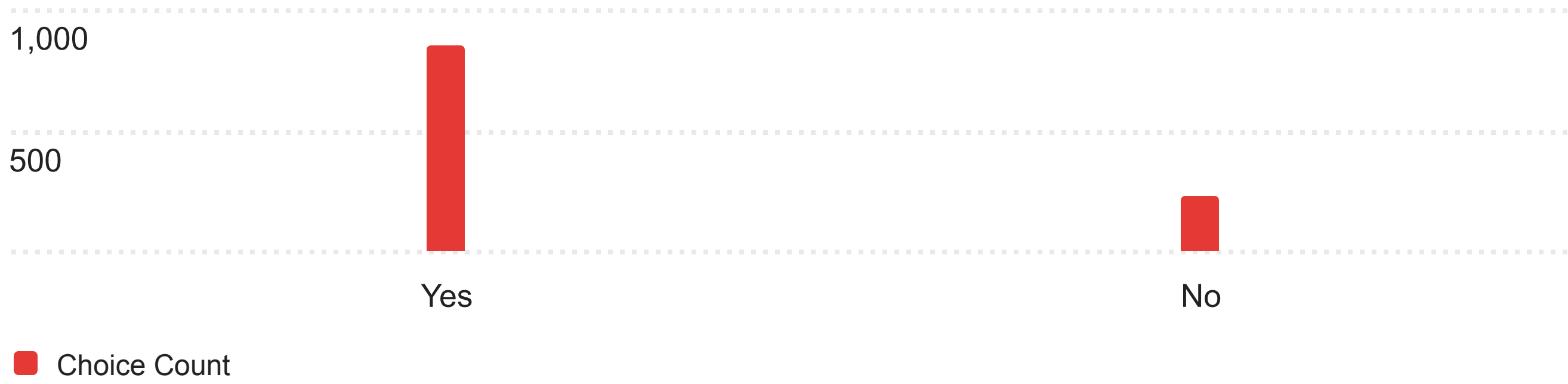

Q12 - What is the greatest number of seizures your pet has had within 24 hours?

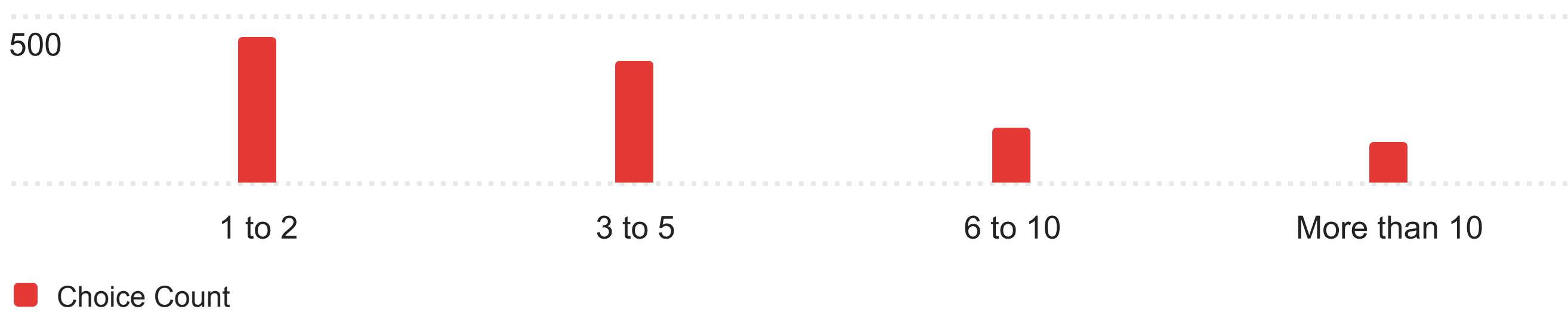

Q13 - Has a veterinarian determined the cause of your pets' seizures? - Selected Choice

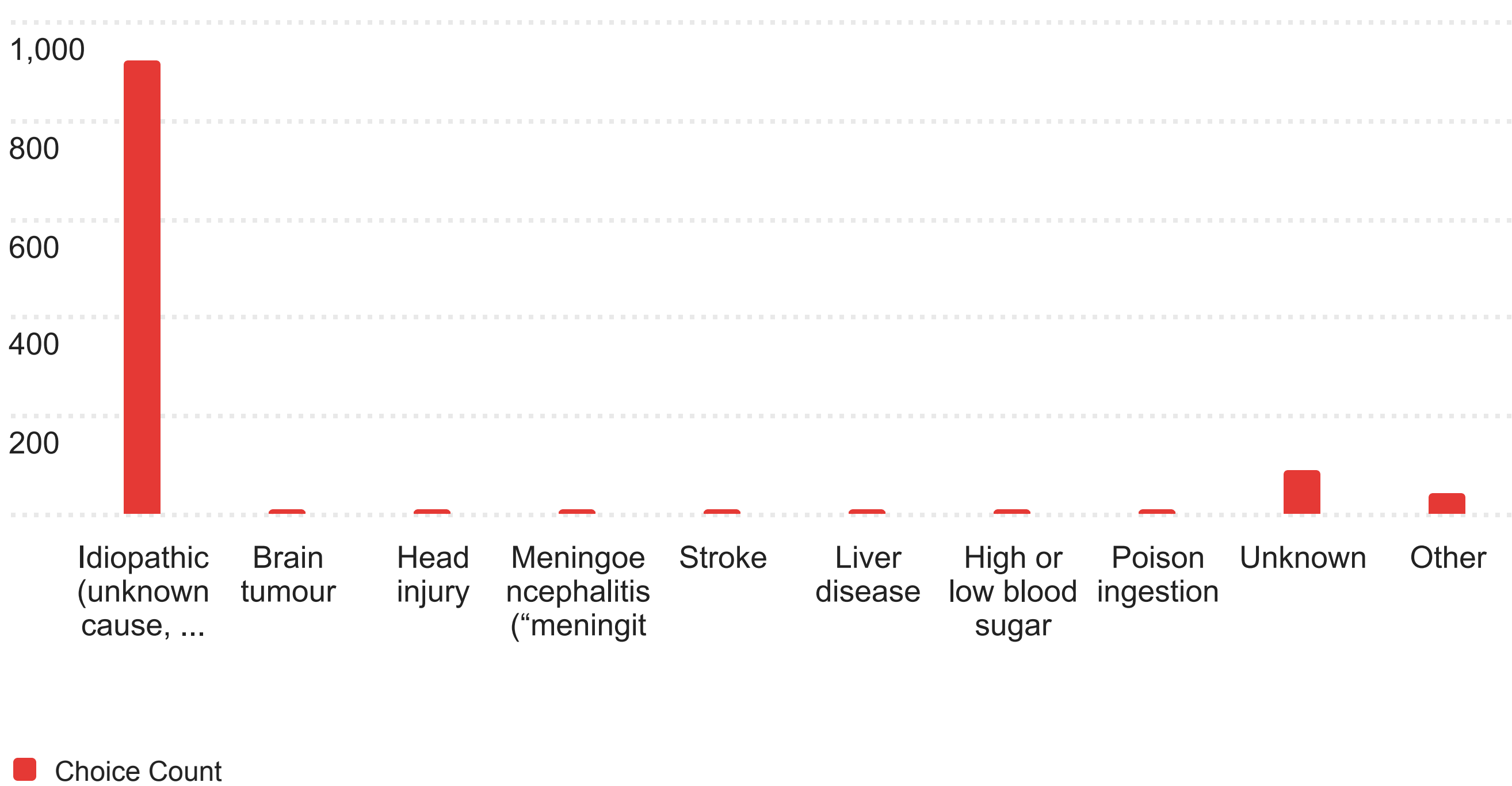

Q14 - Have any of the following tests been done for your pet by a veterinarian (please select all options that apply)? - Selected Choice

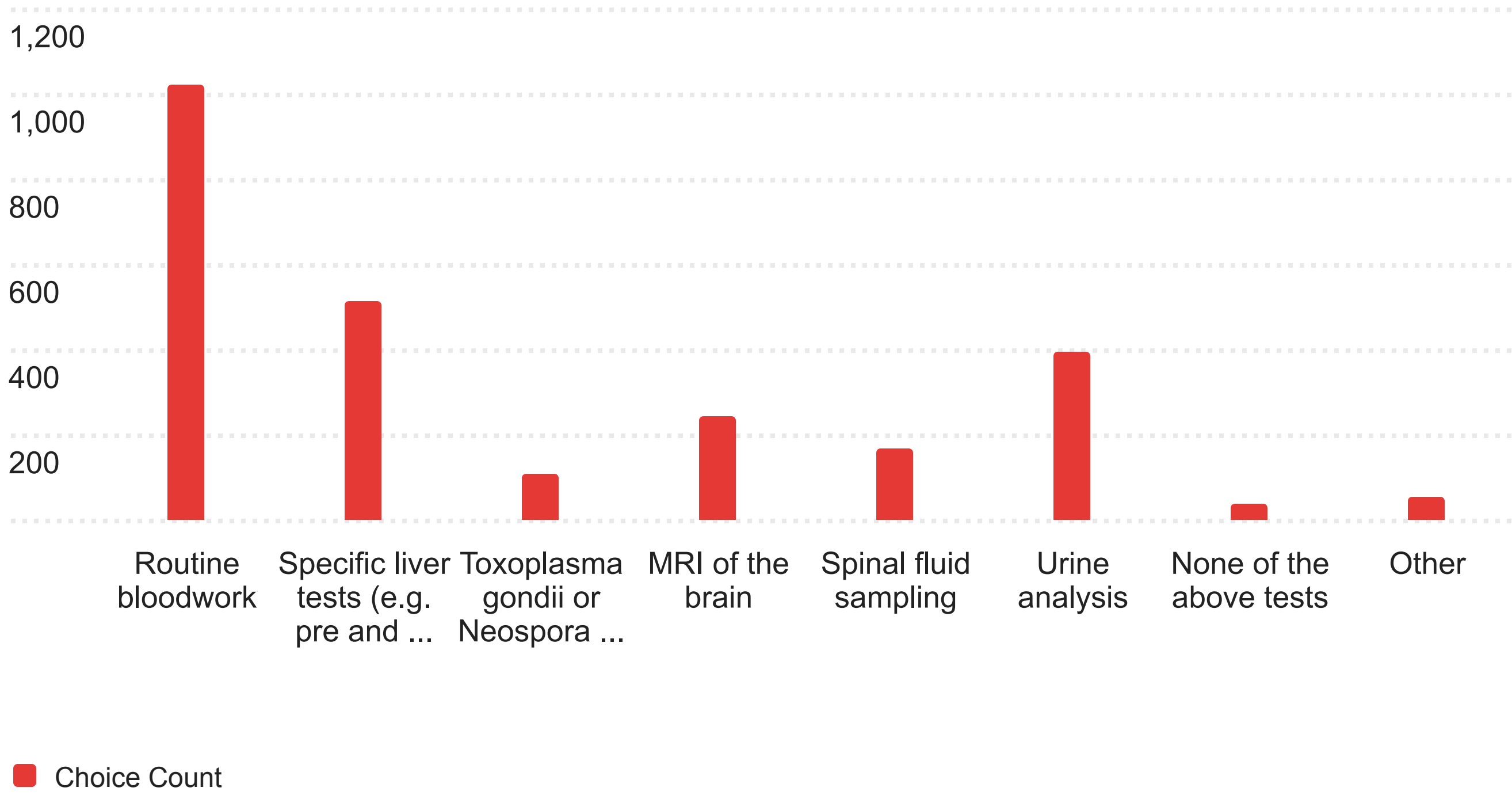

Q15 - Does your pet have any cardiac conditions? If yes, please list them in the comments. - Selected Choice

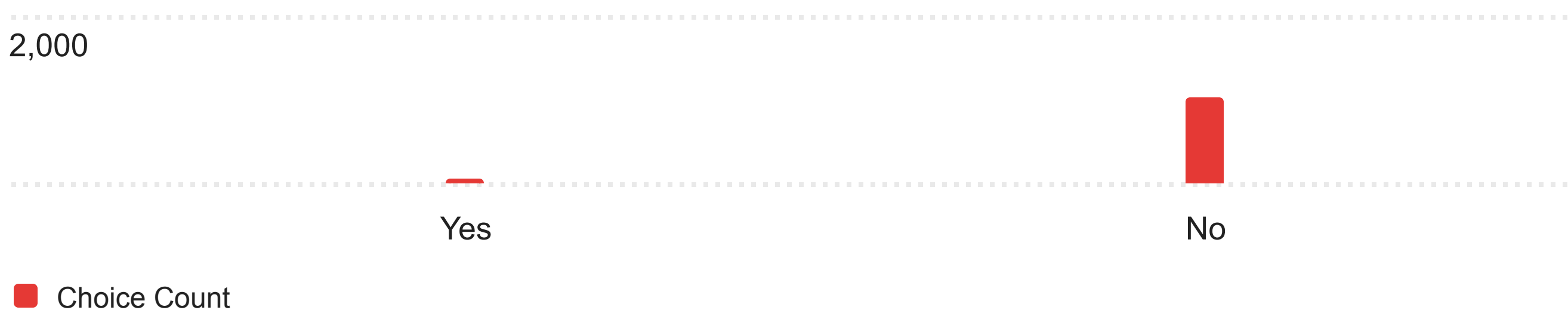

Q16 - Does your pet have any other ongoing health conditions? If yes, please list them in the comments. - Selected Choice

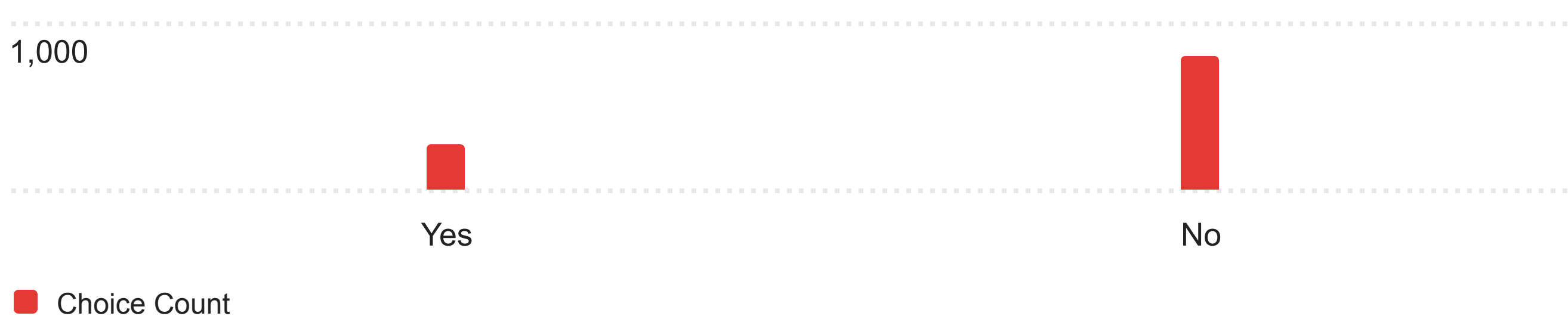

Q17 - Over the last 6 months, how many seizures did your pet have each month on average?

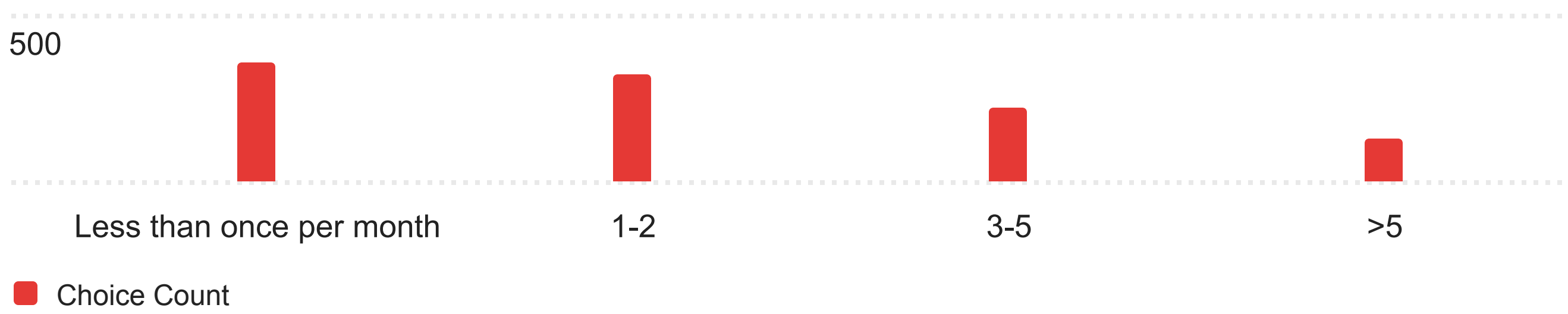

Q18 - If your pet has less than one seizure per month on average, what is the average time interval (in months) between seizures?

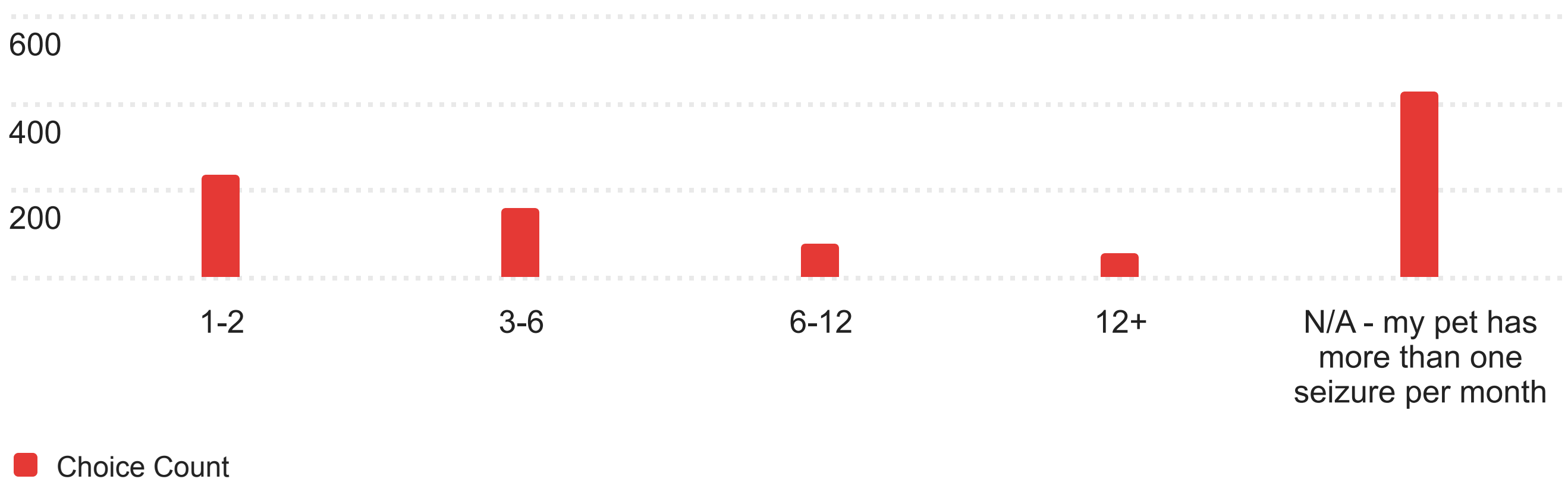

Q19 - What medication(s) is your pet receiving currently for his/her seizures (please select all options that apply)? - Selected Choice

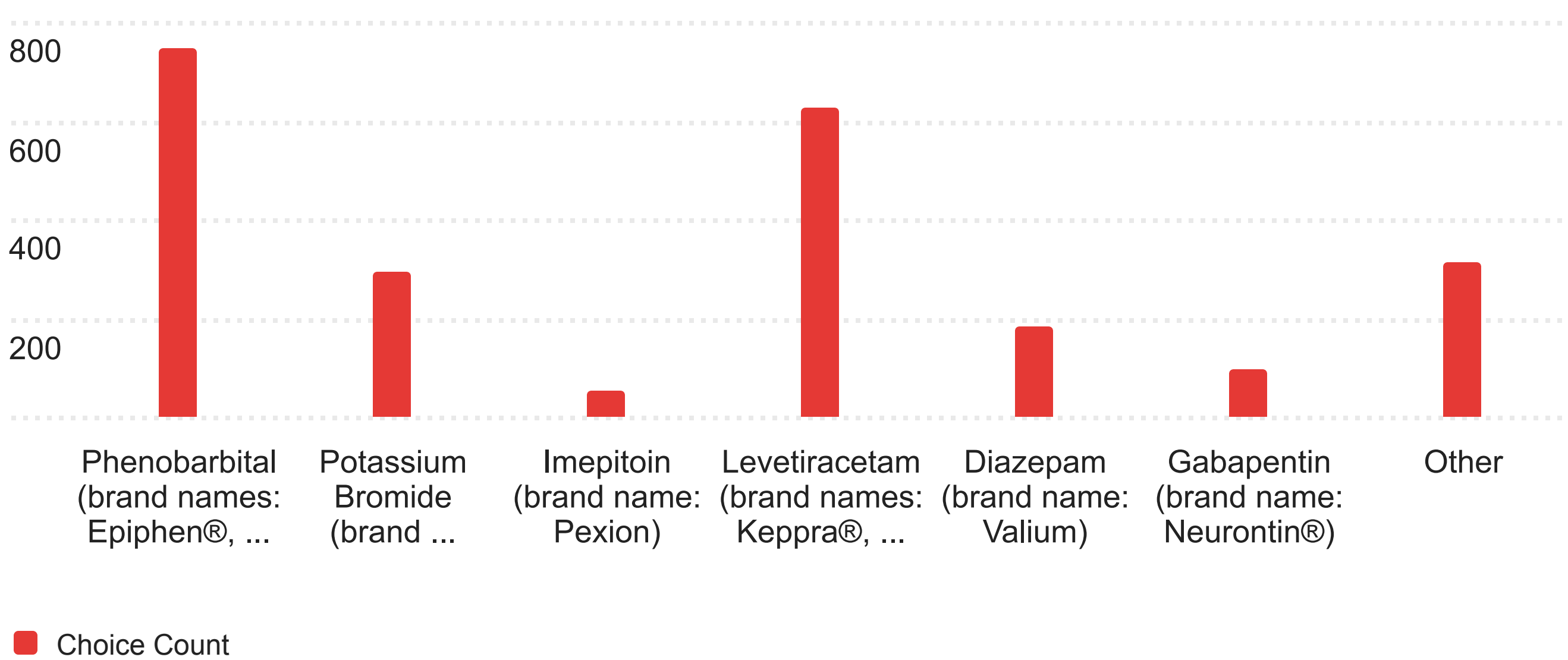

Q20 - How would you rate the typical severity of your dog’s seizures on a scale of 1 to 5?

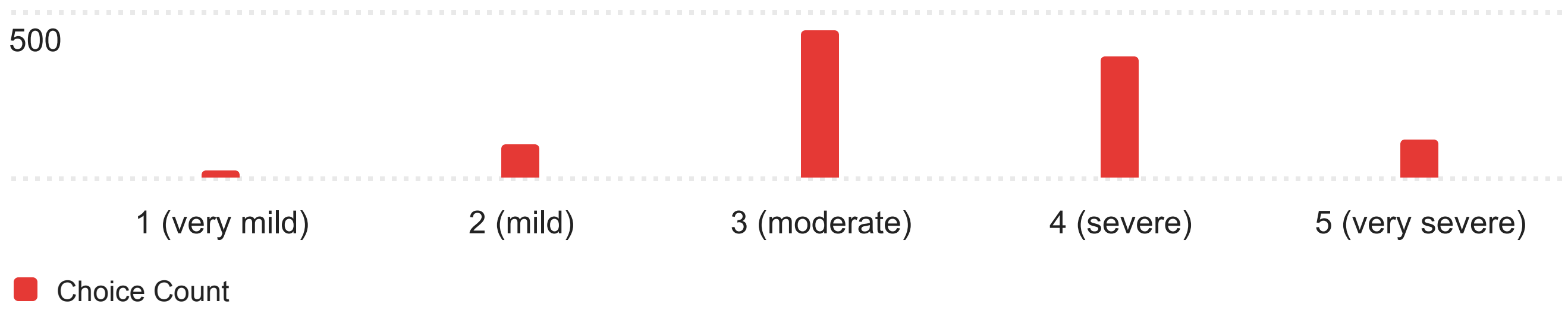

Q21 - How long do your pet's seizures typically last (average duration)?

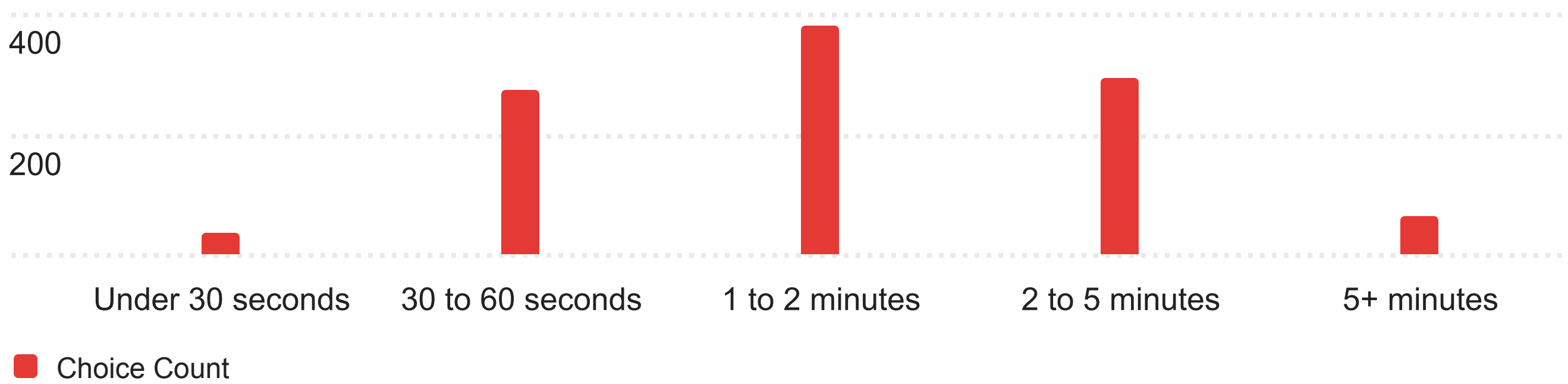

Supplement: Supplementary file 1 [file Data_Sheet_1.pdf]
