# Supplementary material for: Owner reported prevalence of autonomic signs in epileptic dogs
Source: Front Vet Sci. 2026 Sep 3;13:1891856. doi: 10.3389/fvets.2026.1891856 (PMC13582489; doi:10.3389/fvets.2026.1891856)
Supplement: Supplementary file 2 [file Data_Sheet_2.PDF]

Q22 - How long does it typically take for your pet to behave "normally" after a seizure? - Selected Choice

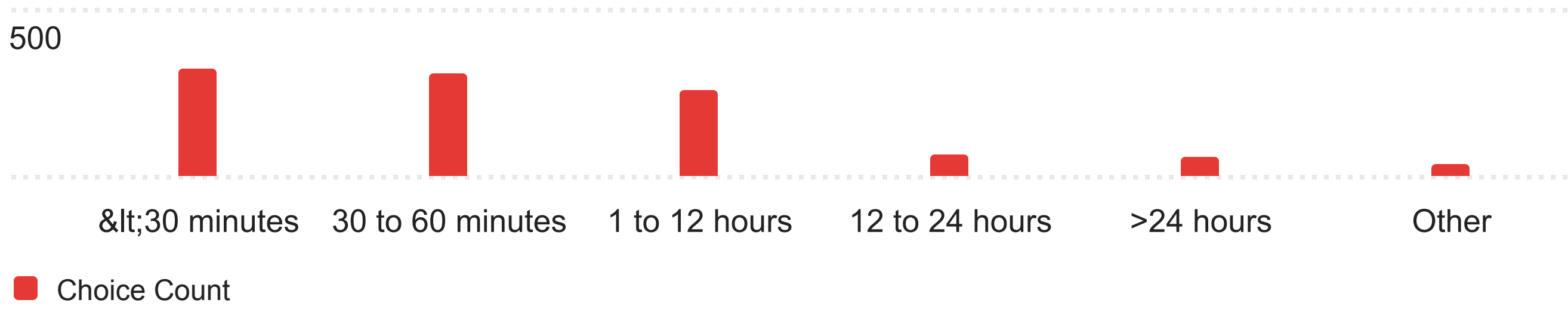

Q23 - How would you describe your pet's normal mood and behaviour (please select all options that apply)?

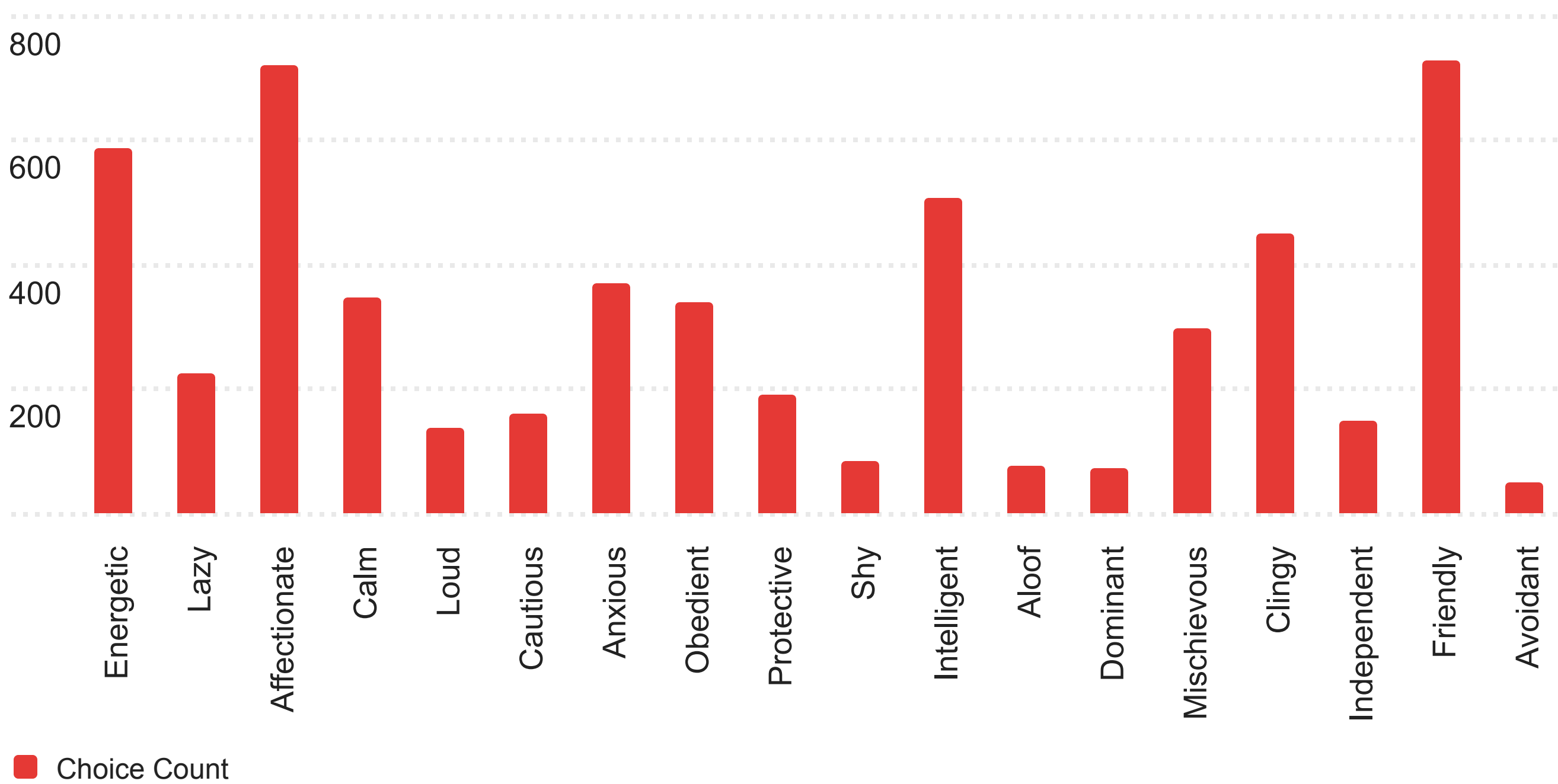

Q24 - How would you describe your pet's mood and behaviour after having a seizure (please select all options that apply)? - Selected Choice

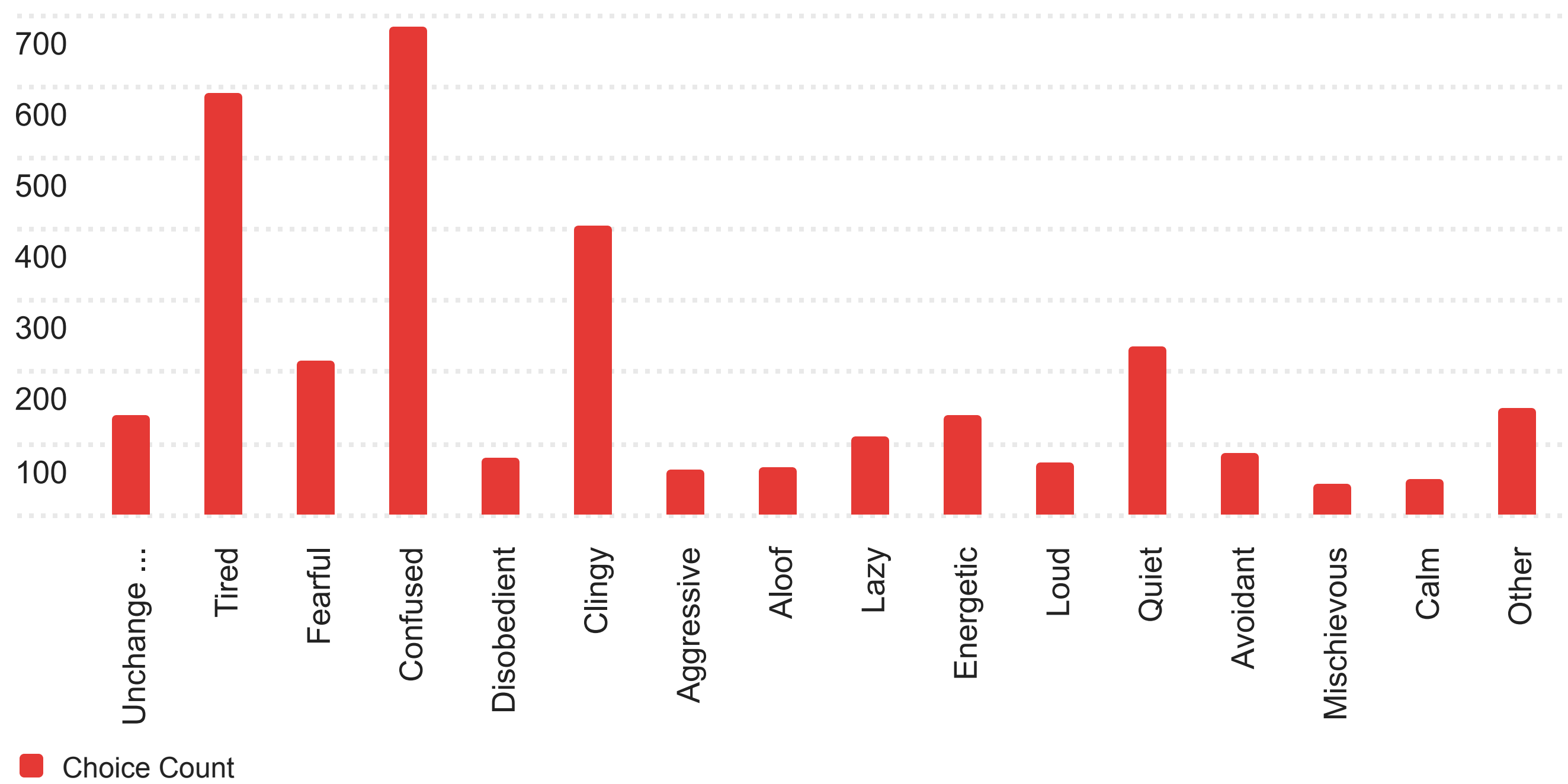

Q25 - How often do each of the following cardiorespiratory signs occur during or...

Field	Min	Max	Mean	Standard Deviation	Variance	Responses	Sum
Hyperventilation (more than 30 breaths per minute)	1.00	6.00	3.71	2.01	4.03	963	3572.00
Hypoventilation (less than 10 breaths per minute)	1.00	6.00	5.34	1.27	1.61	943	5034.00
Apnoea (temporarily stops breathing during sleep)	1.00	6.00	5.69	0.88	0.78	933	5309.00
Coughing	1.00	6.00	5.56	0.92	0.85	936	5207.00
Cyanosis (purple or blue gum colour)	1.00	6.00	5.60	0.98	0.96	930	5208.00
Gagging	1.00	6.00	5.19	1.31	1.70	933	4842.00

Q26 - Have these symptoms changed in severity/frequency over time?

Field	Min	Max	Mean	Standard Deviation	Variance	Responses	Sum
Hyperventilation	1.00	3.00	2.04	0.37	0.14	886	1811.00
Hypoventilation	1.00	3.00	2.00	0.25	0.06	846	1695.00
Apnoea	1.00	3.00	2.00	0.21	0.05	836	1672.00
Coughing	1.00	3.00	2.01	0.25	0.06	840	1691.00
Cyanosis	1.00	3.00	1.99	0.23	0.05	832	1657.00
Gagging	1.00	3.00	2.03	0.30	0.09	845	1716.00

Q27 - How often do each of the following gastrointestinal signs occur during or i...

Field	Min	Max	Mean	Standard Deviation	Variance	Responses	Sum
Ptyalism (excessive drooling)	1.00	6.00	2.52	1.87	3.48	954	2400.00
Appetite changes (temporary around time of seizures)	1.00	6.00	3.33	2.01	4.05	950	3167.00
Abdominal pain	1.00	6.00	5.63	0.89	0.78	936	5266.00
Vomiting	1.00	6.00	5.30	1.33	1.78	942	4992.00
Non-productive retching	1.00	6.00	5.42	1.15	1.33	934	5065.00
Flatulence	1.00	6.00	5.06	1.44	2.08	935	4735.00
Defecation	1.00	6.00	4.51	1.67	2.79	945	4266.00

Q28 - Have these symptoms changed in severity/frequency over time?

Field	Min	Max	Mean	Standard Deviation	Variance	Responses	Sum
Ptyalism	1.00	3.00	2.04	0.32	0.10	901	1839.00
Appetite changes	1.00	3.00	2.05	0.34	0.11	906	1855.00
Abdominal pain	1.00	3.00	1.99	0.18	0.03	878	1746.00

Vomiting	1.00	3.00	1.99	0.28	0.08	888	1767.00
Non-productive retching	1.00	3.00	1.99	0.23	0.05	881	1754.00
Flatulence	1.00	3.00	2.02	0.26	0.07	888	1795.00
Defecation	1.00	3.00	1.95	0.39	0.15	899	1757.00

Q29 - How often do each of the following cutaneous (skin/fur) signs occur during...

Field	Min	Max	Mean	Standard Deviation	Variance	Responses	Sum
Piloerection ("raised hackles" or hair standing on end over spine)	1.00	6.00	5.26	1.37	1.89	924	4856.00
Sweating paws	1.00	6.00	5.45	1.13	1.28	922	5024.00
Pallor (pale skin, may be most noticeable in less hairy skin, e.g. in ears)	1.00	6.00	5.58	0.96	0.92	917	5115.00
Erythema (reddening of skin, may be most noticeable in less hairy skin, e.g. in ears)	1.00	6.00	5.44	1.12	1.26	918	4996.00

Q30 - Have these symptoms changed in severity/frequency over time?

Field	Min	Max	Mean	Standard Deviation	Variance	Responses	Sum
Piloerection	1.00	3.00	1.99	0.14	0.02	863	1716.00
Sweating paws	1.00	3.00	2.00	0.17	0.03	864	1727.00
Pallor	1.00	3.00	1.99	0.15	0.02	861	1716.00
Erythema	1.00	3.00	2.00	0.19	0.04	861	1721.00

Q31 - How often do each of the following ocular (eye) signs occur during or immed...

Field	Min	Max	Mean	Standard Deviation	Variance	Responses	Sum

Mydriasis (dilated pupils)	1.00	6.00	3.46	1.98	3.94	885	3066.00
Miosis (constricted pupils)	1.00	6.00	5.40	1.13	1.28	873	4715.00
Anisocoria (pupils are different sizes)	1.00	6.00	5.64	0.95	0.90	872	4914.00
Lacrimation (increased tear production)	1.00	6.00	5.17	1.36	1.86	872	4507.00

Q32 - Have these symptoms changed in severity/frequency over time?

Field	Min	Max	Mean	Standard Deviation	Variance	Responses	Sum
Mydriasis	1.00	3.00	2.01	0.23	0.05	839	1689.00
Miosis	1.00	3.00	2.00	0.17	0.03	826	1649.00
Anisocoria	1.00	3.00	1.99	0.16	0.03	824	1641.00
Lacrimation	1.00	3.00	2.02	0.24	0.06	825	1668.00

Q33 - How often do each of the following urogenital signs occur during or immedia...

Field	Min	Max	Mean	Standard Deviation	Variance	Responses	Sum
Urination	1.00	6.00	3.17	1.94	3.76	900	2849.00
Polydipsia (excessive water intake within 5 minutes of seizure)	1.00	6.00	3.39	1.86	3.45	897	3038.00
Priapism (persistent erection in males in the absence of any sexual stimuli) (N.B. for females please select never)	2.00	6.00	5.85	0.54	0.30	842	4922.00

Q34 - Have these symptoms changed in severity/frequency over time?

Field	Min	Max	Mean	Standard Deviation	Variance	Responses	Sum
Urination	1.00	3.00	1.99	0.42	0.17	870	1735.00

Polydipsia	1.00	3.00	2.03	0.27	0.07	863	1752.00
Priapism	1.00	3.00	1.98	0.16	0.03	817	1618.00
