# Supplementary material for: Owner reported prevalence of autonomic signs in epileptic dogs
Source: Front Vet Sci. 2026 Sep 3;13:1891856. doi: 10.3389/fvets.2026.1891856 (PMC13582489; doi:10.3389/fvets.2026.1891856)
Supplement: Supplementary file 3 [file Data_Sheet_3.PDF]

ANS Survey 2025

Start of Block: Consent

Q1 I have read and agreed to participate anonymously in this survey.

○ Yes (1)

End of Block: Consent

Start of Block: General Information

Q2 How old is your pet?

○ <1 year (1)

○ 1-6 years (2)

○ 6-10 years (3)

○ 10+ years (4)

Q3 What is your pet's gender?

○ Male (1)

○ Female (2)

Q4 What is your pet's breed?

____

Q5 Has your pet been spayed/neutered/castrated?

◯ Yes (1)

◯ No (2)

Q6 What was the approximate age your pet began having seizures?

◯ < 6 months (1)

◯ 6 to 12 months (2)

◯ 1 to 6 years (3)

◯ 7 to 10 years (4)

◯ over 10 years (5)

Q7 What type of seizures does your pet have (please select all relevant answers)?

▢ Generalized tonic-clonic (whole body involved, loss of consciousness, limbs may twitch, jerk or paddle and are often rigid) (1)

▢ Focal/ partial seizures (usually conscious, not the whole body but only a small area is affected such as twitching and jerking in one side of your pet's body and/or face, dog's trunk curving to one side or head turning to the other) (2)

▢ Absence seizure (difficult to recognize but usually brief lasting for about a minute, e.g. staring, lip licking, eyes rolling) (3)

Q8 Has your pet ever required hospitalisation because of his/her seizures?

◯ Yes (1)

◯ No (2)

Q9 Has your pet had a seizure lasting greater than 5 minutes?

- Yes (1)
- No (2)

Q10 What is the longest a seizure has gone on for?

- <1 minute (1)
- 1-3 minutes (2)
- 3-5 minutes (3)
- 5+ minutes (4)

Q11 Has your pet had two or more seizures within 24 hours?

- Yes (1)
- No (2)

Q12 What is the greatest number of seizures your pet has had within 24 hours?

- 1 to 2 (1)
- 3 to 5 (2)
- 6 to 10 (3)
- More than 10 (4)

End of Block: General Information

Start of Block: Seizure Investigations

Q13 Has a veterinarian determined the cause of your pets' seizures?

- ○ Idiopathic (unknown cause, i.e. "idiopathic epilepsy") (1)
- ○ Brain tumour (2)
- ○ Head injury (3)
- ○ Meningoencephalitis ("meningitis") (4)
- ○ Stroke (5)
- ○ Liver disease (6)
- ○ High or low blood sugar (7)
- ○ Poison ingestion (8)
- ○ Unknown (9)
- ○ Other (10) ____

Q14 Have any of the following tests been done for your pet by a veterinarian (please select all options that apply)?

- ▢ Routine bloodwork (1)
- ▢ Specific liver tests (e.g. pre and post prandial bile acid stimulation test, ammonia) (2)
- ▢ Toxoplasma gondii or Neospora caninum antibody titres (3)
- ▢ MRI of the brain (4)
- ▢ Spinal fluid sampling (5)
- ▢ Urine analysis (6)
- ▢ None of the above tests (7)
- ▢ Other (8) ____

Q15 Does your pet have any cardiac conditions? If yes, please list them in the comments.

- ○ Yes (1) ____
- ○ No (2)

Q16 Does your pet have any other ongoing health conditions? If yes, please list them in the comments.

- ○ Yes (1) ____
- ○ No (2)

End of Block: Seizure Investigations

Start of Block: Seizure Characteristics

Q17 Over the last 6 months, how many seizures did your pet have each month on average?

- Less than once per month (1)
- 1-2 (2)
- 3-5 (3)
- >5 (4)

Q18 If your pet has less than one seizure per month on average, what is the **average** time interval (in months) between seizures?

- 1-2 (1)
- 3-6 (2)
- 6-12 (3)
- 12+ (4)
- N/A - my pet has more than one seizure per month (5)

Q19 What medication(s) is your pet receiving currently for his/her seizures (please select all options that apply)?

- ▢ Phenobarbital (brand names: Epiphen®, Soliphen®, Epityl®) (1)
- ▢ Potassium Bromide (brand names: K-BroVet®, Epilease®, Libromide® also known as KBr) (2)
- ▢ Imepitoin (brand name: Pexion) (3)
- ▢ Levetiracetam (brand names: Keppra®, Elepsia®, Spritam®) (4)
- ▢ Diazepam (brand name: Valium) (5)
- ▢ Gabapentin (brand name: Neurontin®) (6)
- ▢ Other (7) ____

Q20 How would you rate the typical severity of your dog's seizures on a scale of 1 to 5?

- ○ 1 (very mild) (1)
- ○ 2 (mild) (2)
- ○ 3 (moderate) (3)
- ○ 4 (severe) (4)
- ○ 5 (very severe) (5)

Q21 How long do your pet's seizures typically last (average duration)?

○ Under 30 seconds (1)

○ 30 to 60 seconds (2)

○ 1 to 2 minutes (3)

○ 2 to 5 minutes (4)

○ 5+ minutes (5)

Q22 How long does it typically take for your pet to behave "normally" after a seizure?

○ <30 minutes (1)

○ 30 to 60 minutes (2)

○ 1 to 12 hours (3)

○ 12 to 24 hours (4)

○ >24 hours (5)

○ Other (6) ____

End of Block: Seizure Characteristics

Start of Block: Behaviour

Q23 How would you describe your pet's normal mood and behaviour (please select all options that apply)?

- ☐ Energetic (1)
- ☐ Lazy (2)
- ☐ Affectionate (3)
- ☐ Calm (4)
- ☐ Loud (5)
- ☐ Cautious (6)
- ☐ Anxious (7)
- ☐ Obedient (8)
- ☐ Protective (9)
- ☐ Shy (10)
- ☐ Intelligent (11)
- ☐ Aloof (12)
- ☐ Dominant (13)
- ☐ Mischievous (14)
- ☐ Clingy (15)
- ☐ Independent (16)
- ☐ Friendly (17)

☐ Avoidant (18)

Q24 How would you describe your pet's mood and behaviour after having a seizure (please select all options that apply)?

- ▢ Unchanged/normal (1)
- ▢ Tired (2)
- ▢ Fearful (3)
- ▢ Confused (4)
- ▢ Disobedient (5)
- ▢ Clingy (6)
- ▢ Aggressive (7)
- ▢ Aloof (8)
- ▢ Lazy (9)
- ▢ Energetic (10)
- ▢ Loud (11)
- ▢ Quiet (12)
- ▢ Avoidant (13)
- ▢ Mischievous (14)
- ▢ Calm (15)
- ▢ Other (16) ____

End of Block: Behaviour

Start of Block: Autonomic Signs

Q25 How often do each of the following **cardiorespiratory** signs occur during or immediately after having a seizure (please select an answer for each sign)?

	Always (100%) (1)	Most of the time (75-99%) (2)	Often (50-75%) (3)	Sometimes (25-49%) (4)	Uncommonly (10-24%) (5)	Rarely/never (<10%)
Hyperventilation (more than 30 breaths per minute) (1)	○	○	○	○	○	○
Hypoventilation (less than 10 breaths per minute) (2)	○	○	○	○	○	○
Apnoea (temporarily stops breathing during sleep) (3)	○	○	○	○	○	○
Coughing (4)	○	○	○	○	○	○
Cyanosis (purple or blue gum colour) (5)	○	○	○	○	○	○
Gagging (6)	○	○	○	○	○	○

Q26 Have these symptoms changed in severity/frequency over time?

	Better (1)	Stayed the same (2)	Worse (3)
Hyperventilation (1)	o	o	o
Hypoventilation (2)	o	o	o
Apnoea (3)	o	o	o
Coughing (4)	o	o	o
Cyanosis (5)	o	o	o
Gagging (6)	o	o	o

Page Break

Q27 How often do each of the following **gastrointestinal** signs occur during or immediately after having a seizure (please select an answer for each sign)?

	Always (100%) (1)	Most of the time (75-99%) (2)	Often (50-75%) (3)	Sometimes (25-49%) (4)	Uncommonly (10-24%) (5)	Rarely/never (<10%)
Ptyalism (excessive drooling) (1)	○	○	○	○	○	○
Appetite changes (temporary around time of seizures) (2)	○	○	○	○	○	○
Abdominal pain (3)	○	○	○	○	○	○
Vomiting (4)	○	○	○	○	○	○
Non-productive retching (5)	○	○	○	○	○	○
Flatulence (6)	○	○	○	○	○	○
Defecation (7)	○	○	○	○	○	○

Q28 Have these symptoms changed in severity/frequency over time?

	Better (1)	Stayed the same (2)	Worse (3)
Ptyalism (1)	○	○	○
Appetite changes (2)	○	○	○
Abdominal pain (3)	○	○	○
Vomiting (4)	○	○	○
Non-productive retching (5)	○	○	○
Flatulence (6)	○	○	○
Defecation (7)	○	○	○

Page Break

Q29 How often do each of the following **cutaneous** (skin/fur) signs occur during or immediately after having a seizure (please select an answer for each sign)?

	Always (100%) (1)	Most of the time (75-99%) (2)	Often (50-75%) (3)	Sometimes (25-49%) (4)	Uncommonly (10-24%) (5)	Rarely/never (<10%)
Piloerection ("raised hackles" or hair standing on end over spine) (1)	○	○	○	○	○	○
Sweating paws (2)	○	○	○	○	○	○
Pallor (pale skin, may be most noticeable in less hairy skin, e.g. in ears) (3)	○	○	○	○	○	○
Erythema (reddening of skin, may be most noticeable in less hairy skin, e.g. in ears) (4)	○	○	○	○	○	○

Q30 Have these symptoms changed in severity/frequency over time?

	Better (1)	Stayed the same (2)	Worse (3)
Piloerection (1)	○	○	○
Sweating paws (2)	○	○	○
Pallor (3)	○	○	○
Erythema (4)	○	○	○

Page Break

Q31 How often do each of the following **ocular** (eye) signs occur during or immediately after having a seizure (please select an answer for each sign)?

	Always (100%) (1)	Most of the time (75-99%) (2)	Often (50-75%) (3)	Sometimes (25-49%) (4)	Uncommonly (10-24%) (5)	Rarely/never (<10%)
Mydriasis (dilated pupils) (1)	○	○	○	○	○	○
Miosis (constricted pupils) (2)	○	○	○	○	○	○
Anisocoria (pupils are different sizes) (3)	○	○	○	○	○	○
Lacrimation (increased tear production) (4)	○	○	○	○	○	○

Q32 Have these symptoms changed in severity/frequency over time?

	Better (1)	Stayed the same (2)	Worse (3)
Mydriasis (1)	○	○	○
Miosis (2)	○	○	○
Anisocoria (3)	○	○	○
Lacrimation (4)	○	○	○

Q33 How often do each of the following **urogenital** signs occur during or immediately after having a seizure (please select an answer for each sign)?

	Always (100%) (1)	Most of the time (75-99%) (2)	Often (50-75%) (3)	Sometimes (25-49%) (4)	Uncommonly (10-24%) (5)	Rarely/never (<10%)
Urination (1)	○	○	○	○	○	○
Polydipsia (excessive water intake within 5 minutes of seizure) (2)	○	○	○	○	○	○
Priapism (persistent erection in males in the absence of any sexual stimuli) (N.B. for females please select never) (3)	○	○	○	○	○	○

Q34 Have these symptoms changed in severity/frequency over time?

	Better (1)	Stayed the same (2)	Worse (3)
Urination (1)	○	○	○
Polydipsia (2)	○	○	○
Priapism (3)	○	○	○
