# Supplementary material for: Owner reported prevalence of autonomic signs in epileptic dogs
Source: Front Vet Sci. 2026 Sep 3;13:1891856. doi: 10.3389/fvets.2026.1891856 (PMC13582489; doi:10.3389/fvets.2026.1891856)
Supplement: Supplementary file 4 [file Table_1.docx]

Table S1. List of breeds and mixed breeds of dogs included in the study

| Breed | No. of dogs | Type | Body size | Skull shape |
| --- | --- | --- | --- | --- |
| Unknown | 47 | - | - | - |
| Border Collie | 50 | Purebreed | Medium (10-20 kg) |  |
| Labrador Retriever | 43 | Purebreed | Large (20-40 kg) |  |
| Golden Retriever | 39 | Purebreed | Large (20-40 kg) |  |
| French Bulldog | 23 | Purebreed | Medium (10-20 kg) | Brachycephalic |
| German Shepherd | 23 | Purebreed | Large (20-40 kg) |  |
| Siberian Husky | 20 | Purebreed | Large (20-40 kg) |  |
| Australian Shepherd | 17 | Purebreed | Large (20-40 kg) |  |
| American Pit Bull Terrier | 14 | Purebreed | Large (20-40 kg) |  |
| Boston Terrier | 13 | Purebreed | Small (5-10 kg) | Brachycephalic |
| Jack Russell Terrier | 12 | Purebreed | Small (5-10 kg) |  |
| Rottweiler | 11 | Purebreed | Giant (≥40 kg) |  |
| Cocker spaniel | 10 | Purebreed | Medium (10-20 kg) |  |
| Beagle | 9 | Purebreed | Medium (10-20 kg) |  |
| Yorkshire Terrier | 9 | Purebreed | Toy (<5kg) | Brachycephalic |
| German Shorthair Pointer | 8 | Purebreed | Large (20-40 kg) |  |
| Australian Cattle Dog | 8 | Purebreed | Medium (10-20 kg) |  |
| Staffordshire Bull Terrier | 8 | Purebreed | Medium (10-20 kg) |  |
| English Springer Spaniel | 7 | Purebreed | Large (20-40 kg) |  |
| Cane Corso | 7 | Purebreed | Giant (≥40 kg) |  |
| Boxer | 7 | Purebreed | Large (20-40 kg) | Brachycephalic |
| Chihuahua | 7 | Purebreed | Toy (<5kg) | Brachycephalic |
| American Staffordshire Terrier | 6 | Purebreed | Large (20-40 kg) |  |
| Australian Kelpie | 6 | Purebreed | Medium (10-20 kg) |  |
| Springer Spaniel | 6 | Purebreed | Medium (10-20 kg) |  |
| Great Dane | 6 | Purebreed | Giant (≥40 kg) |  |
| Cavalier King Charles Spaniel (CKCS) | 5 | Purebreed | Small (5-10 kg) | Brachycephalic |
| Irish Setter | 5 | Purebreed | Large (20-40 kg) |  |
| Weimaraner | 5 | Purebreed | Large (20-40 kg) |  |
| Pug | 5 | Purebreed | Small (5-10 kg) | Brachycephalic |
| Standard Poodle | 5 | Purebreed | Large (20-40 kg) |  |
| Rhodesian Ridgeback | 5 | Purebreed | Giant (≥40 kg) |  |
| Miniature Dachshund | 5 | Purebreed | Small (5-10 kg) |  |
| English Bulldog | 4 | Purebreed | Large (20-40 kg) | Brachycephalic |
| American Bulldog | 4 | Purebreed | Large (20-40 kg) | Brachycephalic |
| Miniature Australian Shepherd | 4 | Purebreed | Small (5-10 kg) |  |
| Greyhound | 4 | Purebreed | Large (20-40 kg) |  |
| Shetland Sheepdog | 4 | Purebreed | Small (5-10 kg) |  |
| Miniature Pinscher | 4 | Purebreed | Toy (<5kg) |  |
| Dalmatian | 4 | Purebreed | Large (20-40 kg) |  |
| Dachshund | 4 | Purebreed | Small (5-10 kg) |  |
| Italian Spinone | 4 | Purebreed | Large (20-40 kg) |  |
| Catahoula Leopard Dog | 3 | Purebreed | Large (20-40 kg) |  |
| Italian Greyhound | 3 | Purebreed | Small (5-10 kg) |  |
| Belgian Shepherd (Malinois) | 3 | Purebreed | Large (20-40 kg) |  |
| Miniature Schnauzer | 3 | Purebreed | Small (5-10 kg) |  |
| Pomeranian | 3 | Purebreed | Toy (<5kg) |  |
| Chow Chow | 3 | Purebreed | Large (20-40 kg) | Brachycephalic |
| Chesapeake Bay Retriever | 3 | Purebreed | Large (20-40 kg) |  |
| Toy Poodle | 3 | Purebreed | Toy (<5kg) |  |
| Whippet | 3 | Purebreed | Medium (10-20 kg) |  |
| Shih-Tzu | 3 | Purebreed | Small (5-10 kg) | Brachycephalic |
| Curly Coated Retriever | 3 | Purebreed | Large (20-40 kg) |  |
| Maltese | 2 | Purebreed | Small (5-10 kg) |  |
| Welsh Corgi Pembroke | 2 | Purebreed | Medium (10-20 kg) |  |
| Coton de Tulear | 2 | Purebreed | Small (5-10 kg) |  |
| Hungarian Vizsla | 2 | Purebreed | Large (20-40 kg) |  |
| Havanese | 2 | Purebreed | Toy (<5kg) |  |
| Doberman Pinscher | 2 | Purebreed | Large (20-40 kg) |  |
| Dogue de Bordeaux | 2 | Purebreed | Giant (≥40 kg) | Brachycephalic |
| Brittany Spaniel | 2 | Purebreed | Medium (10-20 kg) |  |
| Saint Bernard | 2 | Purebreed | Giant (≥40 kg) |  |
| Alaskan Malamute | 2 | Purebreed | Large (20-40 kg) |  |
| Fox Terrier | 1 | Purebreed | Small (5-10 kg) |  |
| English Setter | 1 | Purebreed | Large (20-40 kg) |  |
| Clumber Spaniel | 1 | Purebreed | Large (20-40 kg) |  |
| Leonberger | 1 | Purebreed | Giant (≥40 kg) |  |
| Petit Bassett Griffin Vendeen | 1 | Purebreed | Medium (10-20 kg) |  |
| Flat Coated Retriever | 1 | Purebreed | Large (20-40 kg) |  |
| Great Pyrenees | 1 | Purebreed | Giant (≥40 kg) |  |
| Boykin spaniel | 1 | Purebreed | Medium (10-20 kg) |  |
| Akita | 1 | Purebreed | Giant (≥40 kg) |  |
| Kokoni | 1 | Purebreed | Small (5-10 kg) |  |
| Scotch Collie (short hair Lassie dog) | 1 | Purebreed | Large (20-40 kg) |  |
| Miniature Poodle | 1 | Purebreed | Small (5-10 kg) |  |
| Japanese Mastiff | 1 | Purebreed | Giant (≥40 kg) | Brachycephalic |
| Old English Sheepdog | 1 | Purebreed | Large (20-40 kg) |  |
| Basset Hound | 1 | Purebreed | Large (20-40 kg) |  |
| Podenco Andaluz | 1 | Purebreed | Large (20-40 kg) |  |
| Bull Terrier | 1 | Purebreed | Medium (10-20 kg) |  |
| Cairn Terrier | 1 | Purebreed | Small (5-10 kg) |  |
| Greater Swiss mountain dog | 1 | Purebreed | Giant (≥40 kg) |  |
| Xoloitzquintli | 1 | Purebreed | Medium (10-20 kg) |  |
| Nova Scotia Duck Tolling Retriever | 1 | Purebreed | Medium (10-20 kg) |  |
| Shiba Inu | 1 | Purebreed | Medium (10-20 kg) |  |
| Bavarian Mountain Scenthound | 1 | Purebreed | Large (20-40 kg) |  |
| Pembroke Corgi | 1 | Purebreed | Small (5-10 kg) |  |
| Rough Collie | 1 | Purebreed | Large (20-40 kg) |  |
| Smooth Collie | 1 | Purebreed | Large (20-40 kg) |  |
| Otterhound | 1 | Purebreed | Large (20-40 kg) |  |
| Belgian Shepherd (Tervuren) | 1 | Purebreed | Large (20-40 kg) |  |
| Scottish Terrier | 1 | Purebreed | Small (5-10 kg) |  |
| American Cocker Spaniel | 1 | Purebreed | Medium (10-20 kg) |  |
| English Cocker Spaniel | 1 | Purebreed | Medium (10-20 kg) |  |
| Giant Schnauzer | 1 | Purebreed | Medium (10-20 kg) |  |
| Old English Bulldog | 1 | Purebreed | Medium (10-20 kg) | Brachycephalic |
| Field Spaniel | 1 | Purebreed | Medium (10-20 kg) |  |
| Parson Russell | 1 | Purebreed | Small (5-10 kg) |  |
| Welsh collie | 1 | Purebreed | Medium (10-20 kg) |  |
| Stabyhoun | 1 | Purebreed | Large (20-40 kg) |  |
| Sussex Spaniel | 1 | Purebreed | Medium (10-20 kg) |  |
| Bloodhound | 1 | Purebreed | Giant (≥40 kg) |  |
| Keeshond | 1 | Purebreed | Medium (10-20 kg) |  |
| Goldendoodle (Labrador Retriever x Poodle) | 11 | Mixed breed | Large (20-40 kg) |  |
| Labradoodle (Labrador Retriever x Poodle) | 9 | Mixed breed | Large (20-40 kg) |  |
| Cockapoo (Cocker Spaniel x Poodle) | 8 | Mixed breed | Medium (10-20 kg) |  |
| Cavapoo (Cavoodle; CKCS x Poodle) | 5 | Mixed breed | Small (5-10 kg) | Brachycephalic |
| American Pit Bull Terrier x Boxer | 3 | Mixed breed | Large (20-40 kg) |  |
| Lurcher (Greyhound or other sighthound crossbreed) | 3 | Mixed breed | Large (20-40 kg) |  |
| Bernedoodle (Bernese Mountain Dog x Poodle) | 2 | Mixed breed | Large (20-40 kg) |  |
| Australian Kelpie x Border Collie | 2 | Mixed breed | Medium (10-20 kg) |  |
| Terrier mix | 2 | Mixed breed | Small (5-10 kg) |  |
| Poochon (Bichon Frise x Poodle) | 2 | Mixed breed | Toy (<5kg) |  |
| Chiweenie (Chihuahua x Dachshund) | 2 | Mixed breed | Toy (<5kg) |  |
| Cavachon (CKCS x Bichon frisé) | 2 | Mixed breed | Small (5-10 kg) |  |
| Border Collie x American Staffordshire Terrier | 1 | Mixed breed | Medium (10-20 kg) |  |
| Boston Terrier x American Staffordshire Terrier | 1 | Mixed breed | Medium (10-20 kg) |  |
| Chihuahua x Fox Terrier | 1 | Mixed breed | Toy (<5kg) |  |
| ShiChi (Shih-tzu x Chihuahua) | 1 | Mixed breed | Small (5-10 kg) | Brachycephalic |
| Border Collie x Whippet | 1 | Mixed breed | Medium (10-20 kg) |  |
| Bernese Mountain Dog x Collie | 1 | Mixed breed | Large (20-40 kg) |  |
| Labrador Retriever x Cocker Spaniel | 1 | Mixed breed | Medium (10-20 kg) |  |
| Jack Russell Terrier x Beagle | 1 | Mixed breed | Small (5-10 kg) |  |
| Pugalier (CKCS x Pug) | 1 | Mixed breed | Small (5-10 kg) | Brachycephalic |
| Alaskan malamute x Golden Retriever | 1 | Mixed breed | Large (20-40 kg) |  |
| Basset Hound x Australian Cattle Dog | 1 | Mixed breed | Large (20-40 kg) |  |
| American Pit Bull Terrier x Labrador Retriever | 1 | Mixed breed | Large (20-40 kg) |  |
| Standard Poodle x Australian shepherd | 1 | Mixed breed | Medium (10-20 kg) |  |
| Basset Hound x German Shepherd | 1 | Mixed breed | Large (20-40 kg) |  |
| Shih-poo (Shih-Tzu x Poodle) | 1 | Mixed breed | Small (5-10 kg) |  |
| Chocolate Labrador Retriever x Great Dane | 1 | Mixed breed | Giant (≥40 kg) |  |
| American Pit Bull Terrier x Poodle | 1 | Mixed breed | Large (20-40 kg) |  |
| Rottweiler x American Bulldog | 1 | Mixed breed | Giant (≥40 kg) |  |
| Labrador Retriever x Australian Cattle Dog x Border Collie | 1 | Mixed breed | Large (20-40 kg) |  |
| Poodle x Bichon Frisé x Yorkshire terrier | 1 | Mixed breed | Small (5-10 kg) |  |
| Scottish Terrier x Miniature Schnauzer | 1 | Mixed breed | Small (5-10 kg) |  |
| Springer Spaniel x Yorkshire Terrier x Shih-tzu | 1 | Mixed breed | Medium (10-20 kg) | Brachycephalic |
| Chihuahua x Welsh Corgi Pembroke | 1 | Mixed breed | Small (5-10 kg) |  |
| Springer Spaniel x Cavapoo | 1 | Mixed breed | Small (5-10 kg) |  |
| American Pit Bull Terrier x Belgian Shepherd | 1 | Mixed breed | Large (20-40 kg) |  |
| Miniature Labradoodle | 1 | Mixed breed | Small (5-10 kg) |  |
| Chihuahua x Yorkshire Terrier | 1 | Mixed breed | Small (5-10 kg) | Brachycephalic |
| Welsh Corgi Pembroke x Australian Cattle Dog | 1 | Mixed breed | Medium (10-20 kg) |  |
| Chihuahua x Terrier | 1 | Mixed breed | Small (5-10 kg) |  |
| French Bulldog x American Staffordshire Terrier | 1 | Mixed breed | Medium (10-20 kg) |  |
| Labrador Retriever x Standard Poodle | 1 | Mixed breed | Large (20-40 kg) |  |
| Maltipoo (Maltese x Poodle) | 1 | Mixed breed | Toy (<5kg) |  |
| Shih-tzu x Bichon Frisé | 1 | Mixed breed | Toy (<5kg) | Brachycephalic |
| Alaskan Malamute x Newfoundland | 1 | Mixed breed | Giant (≥40 kg) |  |
| Standard Poodle x Rottweiler | 1 | Mixed breed | Giant (≥40 kg) |  |
| French Bulldog x Pug | 1 | Mixed breed | Small (5-10 kg) | Brachycephalic |
| Great Pyrenese x Poodle | 1 | Mixed breed | Giant (≥40 kg) |  |
| American Pit Bull Terrier x Chihuahua | 1 | Mixed breed | Medium (10-20 kg) |  |
| Puggle (Pug x Beagle) | 1 | Mixed breed | Small (5-10 kg) |  |
| Border Collie x Welsh Collie | 1 | Mixed breed | Medium (10-20 kg) |  |
| Greek hound mix | 1 | Mixed breed | Medium (10-20 kg) |  |
| Patterjack (Patterdale x Jack Russell Terrier) | 1 | Mixed breed | Small (5-10 kg) |  |
| Dalmatian x Border Collie | 1 | Mixed breed | Medium (10-20 kg) |  |
| Border Collie x Parsons Terrier x Brittany Spaniel | 1 | Mixed breed | Medium (10-20 kg) |  |
| Pomchi (Pomeranian x Chihuahua) | 1 | Mixed breed | Toy (<5kg) | Brachycephalic |
| Boxer x American Bulldog x American Staffordshire Terrier | 1 | Mixed breed | Large (20-40 kg) | Brachycephalic |
| Rottweiler x Labrador Retriever | 1 | Mixed breed | Large (20-40 kg) |  |
| Coltriever (Border Collie x Golden Retriever) | 1 | Mixed breed | Large (20-40 kg) |  |
| Bordoodle (Border Collie x Poodle) | 1 | Mixed breed | Medium (10-20 kg) |  |
| American Staffordshire Terrier x Welsh Corgi Pembroke | 1 | Mixed breed | Medium (10-20 kg) |  |
| Jack Russell Terrier x Lurcher | 1 | Mixed breed | Medium (10-20 kg) |  |
| Border Collie x Miniature Poodle | 1 | Mixed breed | Medium (10-20 kg) |  |
| Saint Berdoodles (Saint Bernard x Standard Poodle) | 1 | Mixed breed | Large (20-40 kg) |  |
| Border Terrier x Jack Russel Terrier | 1 | Mixed breed | Small (5-10 kg) |  |
| American Staffordshire Terrier x Boxer | 1 | Mixed breed | Large (20-40 kg) |  |
| Chihuahua x Pomeranian x Yorkshire terrier | 1 | Mixed breed | Toy (<5kg) | Brachycephalic |
| Springer Spaniel x Whippet x Jack Russell Terrier | 1 | Mixed breed | Medium (10-20 kg) |  |
| Border Collie x Spaniel | 1 | Mixed breed | Medium (10-20 kg) |  |
| Great Pyrenees x Australian Shepherd | 1 | Mixed breed | Large (20-40 kg) |  |
| Pomsky (Siberian Husky x Pomeranian) | 1 | Mixed breed | Small (5-10 kg) |  |
| Siberian Husky x German Shepherd | 1 | Mixed breed | Large (20-40 kg) |  |
| Labrador Retriever x German Shepherd | 1 | Mixed breed | Large (20-40 kg) |  |
| Miniature Pinscher x Italian greyhound | 1 | Mixed breed | Toy (<5kg) |  |
| Miniature Goldendoodle | 1 | Mixed breed | Small (5-10 kg) |  |
| Siberian Husky x Labrador Retriever | 1 | Mixed breed | Large (20-40 kg) |  |
| Labrador Retriever x American Pit Bull | 1 | Mixed breed | Large (20-40 kg) |  |
| Siberian Husky x Belgian Shepherd (Malinois) | 1 | Mixed breed | Large (20-40 kg) |  |
| Labrador Retriever x Shepard | 1 | Mixed breed | Large (20-40 kg) |  |
| Labrador Retriever x Rottweiler | 1 | Mixed breed | Large (20-40 kg) |  |
| Labrador Retriever x Springer Spaniel | 1 | Mixed breed | Large (20-40 kg) |  |
| Cane Corso x Pit Bull | 1 | Mixed breed | Giant (≥40 kg) |  |
| German Shepherd x Pit Bull | 1 | Mixed breed | Large (20-40 kg) |  |
| Bogle (Beagle x Boston Terrier) | 1 | Mixed breed | Medium (10-20 kg) |  |
