# Supplementary material for: Owner reported prevalence of autonomic signs in epileptic dogs
Source: Front Vet Sci. 2026 Sep 3;13:1891856. doi: 10.3389/fvets.2026.1891856 (PMC13582489; doi:10.3389/fvets.2026.1891856)
Supplement: Supplementary file 5 [file Table_2.docx]

Table S2. Demographic and clinical characteristics of dogs. N indicates the number of dogs for which the information was available

| Dog characteristics | Categories | Number (%) of dogs |
| --- | --- | --- |
| Demographic characteristics | | |
| Sex [N=704] | Males  Females | 452 (64.2%)  252 (35.8%) |
| Neuter status [N=704] | Entire  Castrated | 174 (24.7%)  530 (75.3%) |
| Sex and neuter status | Female entire (FE)  Male entire (ME)  Female spayed (FS)  Male neutered (MN) | 47 (6.7%)  127 (18.0%)  205 (29.1%)  325 (46.2%) |
| Age at the moment of completing questionnaire [N=703] | <1 year  1–6 years  7–10 years  >10 years | 10 (1.4%)  418 (59.5%)  202 (28.7%)  73 (10.4%) |
| Breed [N=704] | Unknown  Mixed breed  Pure breed | 47 (6.7%)  124 (17.6%)  533 (75.7%) |
| Skull shape [N=657] | Non-brachycephalic  Brachycephalic | 555 (84.5%)  102 (15.5%) |
| Body size [N=657] | Miniature (<5 kg)  Small (5–10 kg)  Medium (10–20 kg)  Large (20–40 kg)  Giant (>40 kg) | 36 (5.5%)  106 (16.1%)  172 (26.2%)  299 (45.5%)  44 (6.7%) |
| Clinical characteristics | | |
| Age at seizure onset [N=704] | <6 months  6–12 months  1–6 years  7–10 years  >10 years | 40 (5.7%)  102 (14.5%)  501 (71.2%)  50 (7.1%)  11 (1.6%) |
| Seizure type [N=704] | GTCS  GTCS & Focal seizures  GTCS & Focal seizures & Absence seizures  Focal seizures  GTCS & Absence seizures  Focal seizures & Absence seizures  Absence seizures | 427 (60.7%)  134 (19.0%)  64 (9.1%)  38 (5.4%)  31 (4.4%)  8 (1.1%)  2 (0.3%) |
| Seizure type [N=704] | GTCS  Non-GTCS | 656 (93.2%)  48 (6.8%) |
| Seizure etiology [N=704] | Idiopathic epilepsy  Brain tumor  Head injury  Meningitis  Stroke  Abnormal blood glucose  Unknown  Other | 613 (87.1%)  3 (0.4%)  2 (0.3%)  3 (0.4%)  1 (0.1%)  1 (0.1%)  53 (7.5%)  28 (4.0%) |
| Diagnostic tests [N=703] | Routine bloodwork  Liver laboratory tests  *T. gondii* or *N. caninum* antibody titres  MRI of the brain  CSF analysis  Urine analysis  None  Other | 677 (96.3%)  351 (49.9%)  73 (10.4%)  162 (23.0%)  110 (15.7%)  283 (40.3%)  24 (3.4%)  35 (5.0%) |
| Number of seizure episodes / month [N=703] | <1 / month  1-2 / month  3-5 / month  >5 / month | 242 (34.4%)  220 (31.3%)  158 (22.5%)  83 (11.8%) |
| The average time interval (in months) between seizures if the dog has <1 episode / month [N=238] | 1-2 months  3-6 months  7-12 months  >12 months | 81 (34.0%)  72 (30.3%)  49 (20.6%)  36 (15.1%) |
| Antiepileptic medical treatment [N=704] | No  Yes | 55 (7.8%)  649 (92.2%) |
| Antiepileptic drugs used [N=704] | Phenobarbital  Potassium bromide  Imepitoin  Levetiracetam  Gabapentinoids  Benzodiazepines  Zonisamide  Topiramate | 508 (72.2%)  193 (27.4%)  43 (6.1%)  435 (61.8%)  74 (10.5%)  152 (21.6%)  90 (12.8%)  9 (1.3%) |
| Other health issues | Skin and ear conditions  Alimentary system conditions  Joint and spine conditions  Heart conditions  Endocrine conditions  Anxiety  Ocular conditions  Neurological conditions  Cancer  Nephro-urological conditions  Respiratory conditions | 52 (7.4%)  52 (7.4%)  35 (5.0%)  33 (4.7%)  20 (2.8%)  12 (1.7%)  11 (1.6%)  9 (1.3%)  8 (1.1%)  7 (1.0%)  3 (0.4%) |

CSF – cerebrospinal spina fluid; GTCS – general tonic-clonic seizures; MRI – magnetic resonance imaging
