# Supplementary material for: Owner reported prevalence of autonomic signs in epileptic dogs
Source: Front Vet Sci. 2026 Sep 3;13:1891856. doi: 10.3389/fvets.2026.1891856 (PMC13582489; doi:10.3389/fvets.2026.1891856)
Supplement: Supplementary file 6 [file Table_3.docx]

Table S3. Prevalence of groups of autonomic signs in the study population (n=704) and frequency distribution of numbers of reported signs. The number of signs in the first column referred to the number of possible signs within each group.

| Group of autonomic signs (maximum possible number of autonomic signs in parentheses) | Number of dogs affected by at least one sign | Prevalence (95% confidence intervals) [%] | Number (%) of dogs in which a particular number of autonomic signs from a relevant group was reported | | | | | | |
| --- | --- | --- | --- | --- | --- | --- | --- | --- | --- |
|  |  |  | 1 | 2 | 3 | 4 | 5 | 6 | 7 |
| Gastrointestinal (7 signs) | 682 | 96.9 (95.3, 97.9) | 77 (11.3) | 144 (21.1)* | 155 (22.7)* | 136 (19.9)* | 66 (9.7) | 55 (8.1) | 49 (7.2) |
| Urogenital (2 signs in females or 3 signs in males signs) | 641 | 91.1 (88.7, 92.9) | 167 (26.1) | 416 (64.9)* | 58 (9.0) | - | - | - | - |
| Cardiorespiratory (6 signs) | 593 | 84.2 (81.4, 86.7) | 214 (36.1)* | 160 (27.0) | 105 (17.7) | 56 (9.4) | 35 (5.9) | 23 (3.9) | - |
| Ocular (4 signs) | 550 | 78.1 (74.9, 81.0) | 216 (39.3)* | 177 (32.2) | 85 (15.5) | 72 (13.1) | - | - | - |
| Cutaneous (4 signs) | 369 | 52.4 (48.7, 56.1) | 150 (40.7)* | 92 (24.9) | 53 (14.4) | 74 (20.1) | - | - | - |

* modal class
