# Supplementary material for: Owner reported prevalence of autonomic signs in epileptic dogs
Source: Front Vet Sci. 2026 Sep 3;13:1891856. doi: 10.3389/fvets.2026.1891856 (PMC13582489; doi:10.3389/fvets.2026.1891856)
Supplement: Supplementary file 7 [file Table_4.docx]

Table S4: Prevalence and intensity of autonomic signs in the study population

| Autonomic sign | Number of dogs affected / Number of dogs for which the information was available | Prevalence (95% confidence interval) [%] | Number (%) of dogs in which a particular intensity of an autonomic sign was reported | | | | |
| --- | --- | --- | --- | --- | --- | --- | --- |
|  |  |  | Uncommonly (10-24%) | Sometimes (25-49%) | Often (50-75%) | Most of the time (75-99%) | Always (100%) |
| Subgroup of gastrointestinal autonomic signs | | | | | | | |
| Ptyalism | 617 / 704 | 87.6 (85.0, 89.9) | 40 (6.5) | 66 (10.7) | 52 (8.4) | 98 (15.9) | 361 (58.5)* |
| Appetite changes | 520 / 704 | 73.9 (70.5, 77.0) | 62 (11.9) | 77 (14.8) | 70 (13.5) | 108 (20.8) | 203 (39.0)* |
| Defecation | 399 / 704 | 56.7 (53.0, 60.3) | 95 (23.8) | 121 (30.3)* | 66 (16.5) | 60 (15.0) | 57 (14.3) |
| Flatulence | 282 / 704 | 40.1 (36.5, 43.7) | 94 (33.3)* | 88 (31.2)* | 42 (14.9) | 23 (8.2) | 35 (12.4) |
| Vomiting | 209 / 704 | 29.7 (26.4, 33.2) | 87 (41.6)* | 64 (30.6) | 9 (4.3) | 21 (10.0) | 28 (13.4) |
| Non-productive retching | 200 / 704 | 28.4 (25.2, 31.9) | 85 (42.5)* | 66 (33.0) | 15 (7.5) | 18 (9.0) | 16 (8.0) |
| Abdominal pain | 150 / 703 | 21.3 (18.5, 24.5) | 86 (57.3)* | 36 (24.0) | 15 (10.0) | 9 (6.0) | 4 (2.7) |
| Subgroup of urogenital autonomic signs | | | | | | | |
| Urination | 556 / 703 | 79.1 (75.9, 81.9) | 51 (9.2) | 97 (17.4) | 72 (12.9) | 125 (22.5) | 211 (37.9)* |
| Polydipsia | 551 / 703 | 78.4 (75.2, 81.3) | 70 (12.7) | 122 (22.1) | 78 (14.2) | 118 (21.4) | 163 (29.6)* |
| Priapism ^a^ | 66 / 452 | 14.6 (11.6, 18.2) | 36 (54.5)* | 19 (28.8) | 7 (10.6) | 4 (6.1) | 0 |
| Subgroup of cardiorespiratory autonomic signs | | | | | | | |
| Tachypnoea | 483 / 703 | 68.7 (65.2, 72.0) | 68 (14.1) | 93 (19.3) | 67 (13.9) | 99 (20.5) | 156 (32.3)* |
| Gagging | 271 / 702 | 38.6 (35.1, 42.3) | 93 (34.3) | 104 (38.4)* | 26 (9.6) | 30 (11.1) | 18 (6.6) |
| Bradypnoea | 206 / 699 | 29.5 (26.2, 33.0) | 79 (38.3)* | 58 (28.2) | 27 (13.1) | 22 (10.7) | 20 (9.7) |
| Coughing | 165 / 701 | 23.5 (20.5, 26.8) | 62 (37.6) | 73 (44.2)* | 18 (10.9) | 10 (6.1) | 2 (1.2) |
| Cyanosis | 144 / 702 | 20.5 (17.7, 23.7) | 69 (47.9)* | 38 (26.4) | 15 (10.4) | 12 (8.3) | 10 (6.9) |
| Apnoea | 117 / 701 | 16.7 (14.1, 19.6) | 56 (47.9)* | 32 (27.4) | 11 (9.4) | 6 (5.1) | 12 (10.3) |
| Subgroup of ocular autonomic signs | | | | | | | |
| Mydriasis | 505 / 703 | 71.8 (68.4, 75.0) | 49 (9.7) | 97 (19.2) | 86 (17.0) | 86 (17.0) | 187 (37.0)* |
| Lacrimation | 265 / 702 | 37.7 (34.2, 41.4) | 78 (29.4) | 88 (33.2)* | 43 (16.2) | 29 (10.9) | 27 (10.2) |
| Miosis | 211 / 703 | 30.0 (26.7, 33.5) | 94 (44.5)* | 65 (30.8) | 25 (11.8) | 14 (6.6) | 13 (6.2) |
| Anisocoria | 132 / 702 | 18.8 (16.1, 21.9) | 80 (60.6)* | 22 (16.7) | 11 (8.3) | 11 (8.3) | 8 (6.1) |
| Subgroup of cutaneous autonomic signs | | | | | | | |
| Piloerection | 222 / 704 | 31.5 (28.2, 35.1) | 74 (33.3)* | 58 (26.1) | 25 (11.3) | 37 (16.7) | 28 (12.6) |
| Erythema | 202 / 704 | 28.7 (25.5, 32.1) | 79 (39.1)* | 65 (32.2) | 23 (11.4) | 20 (9.9) | 15 (7.4) |
| Sweating paws | 193 / 704 | 27.4 (24.2, 30.8) | 85 (44.0)* | 49 (25.4) | 26 (13.5) | 22 (11.4) | 11 (5.7) |
| Pallor | 172 / 703 | 24.5 (21.4, 27.8) | 85 (49.4)* | 52 (30.2) | 16 (9.3) | 11 (6.4) | 8 (4.7) |

* modal class

^a^ autonomic sign reported only for males
