# Supplementary material for: Owner reported prevalence of autonomic signs in epileptic dogs
Source: Front Vet Sci. 2026 Sep 3;13:1891856. doi: 10.3389/fvets.2026.1891856 (PMC13582489; doi:10.3389/fvets.2026.1891856)
Supplement: Supplementary file 8 [file Table_5.docx]

Table S5. Univariable analysis of association between demographic and clinical characteristics of dogs and the presence of gastrointestinal autonomic signs. N indicates the number of dogs for which the information was available

| Dog characteristics | Category | No. (%) of dogs with gastrointestinal autonomic signs in the category | p-value ^c^ | Odds ratio (95% confidence interval) |
| --- | --- | --- | --- | --- |
| Demographic characteristics | | | | |
| Sex | Females | 241 / 252 (95.6%) | 0.167 | Reference category |
|  | Males | 441 / 452 (97.6%) |  | 1.83 (0.78, 4.28) |
| Neuter status | Entire | 168 / 174 (96.6%) | 0.780 | Reference category |
|  | Neutered | 514 / 530 (97.0%) |  | 1.15 (0.44, 2.98) |
| Body size | Miniature (<5 kg) | 33 / 36 (91.7%) | 0.154 | Reference category |
|  | Small (5-10 kg) | 102 / 106 (96.2%) | 0.301 | 2.32 (0.49, 10.9) |
|  | Medium (10-20 kg) | 164 / 172 (95.4%) | 0.397 | 1.86 (0.47, 7.40) |
|  | Large (20-40 kg) | 293 / 299 (98.0%) | 0.064 | 4.44 (1.06, 18.6) |
|  | Giant (>40 kg) | 43 / 44 (97.7%) | 0.211 | 3.91 (0.39, 39.3) |
|  | Unknown | 47 / 47 (100%) | 0.023 | - |
| Skull shape | Non-brachycephalic | 539 / 555 (97.1%) | 0.077 | Reference category |
|  | Brachycephalic | 96 / 102 (94.1%) | 0.152 | 0.48 (0.18, 1.24) |
|  | Unknown | 47 / 47 (100%) | 0.104 | - |
| Border collie | No | 634 / 654 (96.9%) | 0.722 | Reference category |
|  | Yes | 48 / 50 (96.0%) |  | 0.76 (0.17, 3.34) |
| Labrador Retriever | No | 640 / 661 (96.8%) | 0.746 | Reference category |
|  | Yes | 42 / 43 (97.7%) |  | 1.38 (0.18, 10.5) |
| Golden Retriever | No | 644 / 665 (96.8%) | 0.831 | Reference category |
|  | Yes | 38 / 39 (97.4%) |  | 1.24 (0.16, 9.46) |
| Age | <6 years | 416 / 428 (97.2%) | 0.803 | Reference category |
|  | 6–10 years | 195 / 202 (96.5%) | 0.651 | 0.80 (0.31, 2.07) |
|  | >10 years | 70 / 73 (95.9%) | 0.548 | 0.67 (0.19, 2.45) |
| Clinical characteristics | | | | |
| Age at which seizures started | <1 year | 137 / 142 (96.5%) | 0.131 | Reference category |
|  | 1–6 years | 484 / 501 (96.6%) | 0.941 | 1.04 (0.38, 2.87) |
|  | >6 years | 61 / 61 (100%) | 0.997 | 4.92 (0.27, 90.4) |
| Seizure type ^a^ | Non-GTCS | 43 / 48 (89.6%) | 0.015* | Reference category |
|  | GTCS | 639 / 656 (97.4%) |  | 4.37 (1.54, 12.4) |
| Number of seizure episodes / month [N=703] ^b^ | <1 / month | 229 / 242 (94.6%) | 0.069 | Reference category |
|  | 1-2 / month | 214 / 220 (97.3%) | 0.161 | 2.03 (0.76, 5.42) |
|  | 3-5 / month | 156 / 158 (98.7%) | 0.052 | 4.43 (0.99, 19.9) |
|  | >5 / month | 82 / 83 (98.8%) | 0.141 | 4.66 (0.60, 36.1) |
| Seizure frequency ^a^ | <1 / month | 229 / 242 (94.6%) | 0.017* | Reference category |
|  | >1 / month | 452 / 461 (98.1%) |  | 2.85 (1.20, 6.77) |
| Any antiepileptic treatment ^a^ | No | 49 / 55 (89.1%) | 0.005* | Reference category |
|  | Yes | 633 / 649 (97.5%) |  | 4.84 (1.81, 12.94) |
| Phenobarbital ^a^ | No | 185 / 196 (94.4%) | 0.026* | Reference category |
|  | Yes | 497 / 508 (97.8%) |  | 2.69 (1.15, 6.30) |
| Potassium bromide | No | 493 / 511 (96.5%) | 0.304 | Reference category |
|  | Yes | 189 / 193 (97.9%) |  | 1.73 (0.58, 5.16) |
| Imepitoin | No | 641 / 661 (97%) | 0.577 | Reference category |
|  | Yes | 41 / 43 (95.3%) |  | 0.64 (0.14, 2.83) |
| Levetiracetam ^a^ | No | 255 / 269 (94.8%) | 0.014* | Reference category |
|  | Yes | 427 / 435 (98.2%) |  | 2.93 (1.21, 7.08) |
| Gabapentinoids | No | 609 / 630 (96.7%) | 0.303 | Reference category |
|  | Yes | 73 / 74 (98.6%) |  | 2.52 (0.33, 19.0) |
| Benzodiazepines | No | 534 / 552 (96.7%) | 0.687 | Reference category |
|  | Yes | 148 / 152 (97.4%) |  | 1.25 (0.42, 3.74) |
| Zonisamide | No | 594 / 614 (96.7%) | 0.581 | Reference category |
|  | Yes | 88 / 90 (97.8%) |  | 1.48 (0.34, 6.45) |
| Topiramate | No | 674 / 695 (97.0%) | 0.276 | Reference category |
|  | Yes | 8 / 9 (88.9%) |  | 0.25 (0.03, 2.08) |
| Alimentary system conditions | No | 631 / 652 (96.8%) | 0.580 | Reference category |
|  | Yes | 51 / 52 (98.1%) |  | 1.70 (0.22, 12.9) |

^a^ included in the multivariable analysis based on p<0.15

^b^ turned into binary variable for inclusion in the multivariable model

^c^ in the case of multi-categorical variables, p-values located at the level of reference category and adjusted to the left side of the column indicate the result of the overall maximum likelihood G test while p-values adjusted to the right side of the column indicate results of comparison of the category at whose level they are located with the reference category

* significant at α=0.05

Table S6. Multivariable analysis of association between demographic and clinical characteristics of dogs and the presence of gastrointestinal autonomic signs

| Variables | Regression coefficient (standard error) | Wald's statistic | p-value | Adjusted odds ratio  (95% confidence interval) |
| --- | --- | --- | --- | --- |
| Intercept | 1.29 (0.56) | - | - | - |
| General tonic-clonic seizures (GTCS) |  | | | |
| Not observed | Reference category | - | - | 1.00 |
| Observed | 1.16 (0.56) | 4.22 | 0.040* | 3.18 (1.06, 9.56) |
| Any antiepileptic medical treatment |  | | | |
| Not used | Reference category | - | - | 1.00 |
| Used | 1.34 (0.53) | 6.46 | 0.011* | 3.81 (1.36, 10.7) |

* significant at α=0.05

Table S7. Univariable analysis of association between demographic and clinical characteristics of dogs and the presence of urogenital autonomic signs. N indicates the number of dogs for which the information was available

| Dog characteristics | Category | No. (%) of dogs with urogenital autonomic signs in the category | p-value ^c^ | Odds ratio (95% confidence interval) |  |
| --- | --- | --- | --- | --- | --- |
| Demographic characteristics | | | | | |
| Sex ^a^ | Females | 221 / 252 (87.7%) | 0.022* | Reference category |  |
|  | Males | 420 / 452 (92.9%) |  | 1.84 (1.09, 3.1) |  |
| Neuter status ^a^ | Entire | 165 / 174 (94.8%) | 0.034* | Reference category |  |
|  | Neutered | 476 / 530 (89.8%) |  | 0.48 (0.23, 1.00) |  |
| Sex & Neuter status ^b^ | ME | 125 / 127 (98.4%) | 0.011* | Reference category |  |
|  | MN | 295 / 325 (90.8%) | 0.001* | 0.16 (0.04, 0.67) |  |
|  | FN | 181 / 205 (88.3%) | <0.001* | 0.12 (0.03, 0.52) |  |
|  | FE | 40 / 47 (85.1%) | <0.001* | 0.09 (0.02, 0.46) |  |
| Sex & Neuter status ^a^ | Female or MN | 516 / 577 (89.4%) | <0.001* | Reference category |  |
|  | ME | 125 / 127 (98.4%) |  | 7.39 (1.78, 30.6) |  |
| Body size | Miniature (<5 kg) | 32 / 36 (88.9%) | 0.581 | Reference category |  |
|  | Small (5-10 kg) | 93 / 106 (87.7%) | 0.853 | 0.89 (0.27, 2.94) |  |
|  | Medium (10-20 kg) | 160 / 172 (93.0%) | 0.419 | 1.67 (0.51, 5.50) |  |
|  | Large (20-40 kg) | 272 / 299 (91.0%) | 0.691 | 1.26 (0.41, 3.83) |  |
|  | Giant (>40 kg) | 42 / 44 (95.5%) | 0.267 | 2.63 (0.45, 15.2) |  |
|  | Unknown | 42 / 47 (89.4%) | 0.945 | - |  |
| Skull shape | Non-brachycephalic | 508 / 555 (91.5%) | 0.700 | Reference category |  |
|  | Brachycephalic | 91 / 102 (89.2%) | 0.460 | 0.77 (0.38, 1.53) |  |
|  | Unknown | 42 / 47 (89.4%) | 0.622 | - |  |
| Border collie | No | 593 / 654 (90.7%) | 0.160 | Reference category |  |
|  | Yes | 48 / 50 (96.0%) |  | 2.47 (0.59, 10.4) |  |
| Labrador Retriever ^a^ | No | 605 / 661 (91.5%) | 0.112 | Reference category |  |
|  | Yes | 36 / 43 (83.7%) |  | 0.48 (0.20, 1.12) |  |
| Golden Retriever | No | 604 / 665 (90.8%) | 0.355 | Reference category |  |
|  | Yes | 37 / 39 (94.9%) |  | 1.87 (0.44, 7.94) |  |
| Age ^b^ | <6 years | 392 / 428 (91.6%) | 0.033* | Reference category |  |
|  | 6–10 years | 188 / 202 (93.1%) | 0.522 | 1.23 (0.65, 2.34) |  |
|  | >10 years | 60 / 73 (82.2%) | 0.015 | 0.42 (0.21, 0.85) |  |
| Age ^a^ | ≤10 years | 580 / 630 (92.1%) | 0.011* | Reference category |  |
|  | >10 years | 60 / 73 (82.2%) |  | 0.40 (0.21, 0.77) |  |
| Clinical characteristics | | | | | |
| Age at which seizures started | <1 year | 127 / 142 (89.4%) | 0.708 | Reference category |  |
|  | 1–6 years | 459 / 501 (91.6%) | 0.429 | 1.29 (0.69, 2.40) |  |
|  | >6 years | 55 / 61 (90.2%) | 0.876 | 1.08 (0.40, 2.94) |  |
| Seizure type ^a^ | Non-GTCS | 36 / 48 (75.0%) | 0.001* | Reference category |  |
|  | GTCS | 605 / 656 (92.2%) |  | 3.95 (1.94, 8.07) |  |
| Number of seizure episodes / month [N=703] | <1 / month | 215 / 242 (88.8%) | 0.285 | Reference category |  |
|  | 1-2 / month | 201 / 220 (91.4%) | 0.367 | 1.33 (0.72, 2.46) |  |
|  | 3-5 / month | 146 / 158 (92.4%) | 0.243 | 1.53 (0.75, 3.11) |  |
|  | >5 / month | 79 / 83 (95.2%) | 0.100 | 2.48 (0.84, 7.31) |  |
| Seizure frequency ^a^ | <1 / month | 215 / 242 (88.8%) | 0.119 | Reference category |  |
|  | >1 / month | 426 / 461 (92.4%) |  | 1.53 (0.90, 2.59) |  |
| Any antiepileptic treatment ^a^ | No | 46 / 55 (83.6%) | 0.066 | Reference category |  |
|  | Yes | 595 / 649 (91.7%) |  | 2.16 (1.00, 4.64) |  |
| Phenobarbital ^a^ | No | 170 / 196 (86.7%) | 0.016* | Reference category |  |
|  | Yes | 471 / 508 (92.7%) |  | 1.95 (1.14, 3.31) |  |
| Potassium bromide ^a^ | No | 459 / 511 (89.8%) | 0.053 | Reference category |  |
|  | Yes | 182 / 193 (94.3%) |  | 1.87 (0.96, 3.67) |  |
| Imepitoin ^a^ | No | 599 / 661 (90.6%) | 0.067 | Reference category |  |
|  | Yes | 42 / 43 (97.7%) |  | 4.35 (0.59, 32.1) |  |
| Levetiracetam ^a^ | No | 239 / 269 (88.8%) | 0.111 | Reference category |  |
|  | Yes | 402 / 435 (92.4%) |  | 1.53 (0.91, 2.57) |  |
| Gabapentinoids | No | 574 / 630 (91.1%) | 0.872 | Reference category |  |
|  | Yes | 67 / 74 (90.5%) |  | 0.93 (0.41, 2.13) |  |
| Benzodiazepines | No | 501 / 552 (90.8%) | 0.602 | Reference category |  |
|  | Yes | 140 / 152 (92.1%) |  | 1.19 (0.62, 2.29) |  |
| Zonisamide | No | 560 / 614 (91.2%) | 0.712 | Reference category |  |
|  | Yes | 81 / 90 (90.0%) |  | 0.87 (0.41, 1.82) |  |
| Topiramate | No | 633 / 695 (91.1%) | 0.825 | Reference category |  |
|  | Yes | 8 / 9 (88.9%) |  | 0.78 (0.10, 6.37) |  |
| Nephro-urological conditions | No | 634 / 697 (98.9%) | 0.250 | Reference category |  |
|  | Yes | 7 / 7 (100%) |  | - |  |

^a^ included in the multivariable analysis based on p<0.15

^b^ turned into binary variable for inclusion in the multivariable model

^c^ in the case of multi-categorical variables, p-values located at the level of reference category and adjusted to the left side of the column indicate the result of the overall maximum likelihood G test while p-values adjusted to the right side of the column indicate results of comparison of the category at whose level they are located with the reference category

* significant at α=0.05

Table S8. Multivariable analysis of association between demographic and clinical characteristics of dogs and the presence of urogenital autonomic signs

| Variables | Regression coefficient (standard error) | Wald's statistic | p-value | Adjusted odds ratio  (95% confidence interval) |
| --- | --- | --- | --- | --- |
| Intercept | 1.19 (0.34) | - | - | - |
| Age |  | | | |
| ≤10 years | Reference category | - | - | 1.00 |
| >10 years | -0.89 (0.35) | 6.43 | 0.011* | 0.41 (0.21, 0.82) |
| Sex & neuter status |  | | | |
| Females or entire males | Reference category | - | - | 1.00 |
| Entire males | 1.83 (0.73) | 6.30 | 0.012* | 6.23 (1.49, 26.0) |
| General tonic-clonic seizures (GTCS) |  | | | |
| Not observed | Reference category | - | - | 1.00 |
| Observed | 1.25 (0.37) | 11.3 | 0.001* | 3.49 (1.69, 7.21) |

Hosmer-Lemeshow χ^2^ test: p=0.756; Nagelkerke’s pseudo-R^2^ coefficient = 0.092; BIC = 415.9

* significant at α=0.05

Table S9. Univariable analysis of association between demographic and clinical characteristics of dogs and the presence of cardiorespiratory autonomic signs. N indicates the number of dogs for which the information was available

| Dog characteristics | Category | No. (%) of dogs with cardiorespiratory autonomic signs in the category | p-value ^c^ | Odds ratio (95% confidence interval) |  |
| --- | --- | --- | --- | --- | --- |
| Demographic characteristics | | | | | |
| Sex | Females | 210 / 252 (83.3%) | 0.626 | Reference category |  |
|  | Males | 383 / 452 (84.7%) |  | 1.11 (0.73, 1.69) |  |
| Neuter status | Entire | 152 / 174 (87.4%) | 0.184 | Reference category |  |
|  | Neutered | 441 / 530 (83.2%) |  | 0.72 (0.43, 1.18) |  |
| Body size ^b^ | Miniature (<5 kg) | 27 / 36 (75.0%) | 0.036* | Reference category |  |
|  | Small (5-10 kg) | 82 / 106 (77.4%) | 0.773 | 1.14 (0.47, 2.75) |  |
|  | Medium (10-20 kg) | 145 / 172 (84.3%) | 0.197 | 1.79 (0.76, 4.23) |  |
|  | Large (20-40 kg) | 261 / 299 (87.3%) | 0.062 | 2.29 (1.00, 5.24) |  |
|  | Giant (>40 kg) | 41 / 44 (93.2%) | 0.022 | 4.56 (1.13, 18.4) |  |
|  | Unknown | 37 / 47 (78.7%) | 0.689 | - |  |
| Body size ^a^ | Miniature or Small | 109 / 142 (76.8%) | 0.011* | Reference category |  |
|  | Medium, Large or Giant | 447 / 515 (86.8%) | 0.004 | 1.99 (1.25, 3.17) |  |
|  | Unknown | 37 / 47 (78.7%) | 0.781 | 1.12 (0.50, 2.49) |  |
| Skull shape | Non-brachycephalic | 468 / 555 (84.3%) | 0.516 | Reference category |  |
|  | Brachycephalic | 88 / 102 (86.3%) | 0.611 | 1.17 (0.64, 2.15) |  |
|  | Unknown | 37 / 47 (78.7%) | 0.333 | - |  |
| Border collie | No | 552 / 654 (84.4%) | 0.658 | Reference category |  |
|  | Yes | 41 / 50 (82.0%) |  | 0.84 (0.40, 1.79) |  |
| Labrador Retriever ^a^ | No | 552 / 661 (83.5%) | 0.019* | Reference category |  |
|  | Yes | 41 / 43 (95.3%) |  | 4.05 (0.96, 17.0) |  |
| Golden Retriever | No | 560 / 665 (84.2%) | 0.946 | Reference category |  |
|  | Yes | 33 / 39 (84.6%) |  | 1.03 (0.42, 2.52) |  |
| Age | <6 years | 367 / 428 (85.7%) | 0.381 | Reference category |  |
|  | 6–10 years | 165 / 202 (81.7%) | 0.741 | 0.74 (0.47, 1.16) |  |
|  | >10 years | 60 / 73 (82.2%) | 0.430 | 0.77 (0.40, 1.48) |  |
| Clinical characteristics | | | | | |
| Age at which seizures started | <1 year | 127 / 142 (89.4%) | 0.061 | Reference category |  |
|  | 1–6 years | 412 / 501 (82.2%) | 0.042 | 0.55 (0.31, 0.98) |  |
|  | >6 years | 54 / 61 (88.5%) | 0.848 | 0.91 (0.35, 2.36) |  |
| Seizure type ^a^ | Non-GTCS | 32 / 48 (66.7%) | 0.002* | Reference category |  |
|  | GTCS | 561 / 656 (85.5%) |  | 2.95 (1.56, 5.59) |  |
| Number of seizure episodes / month [N=703] ^b^ | <1 / month | 192 / 242 (79.3%) | 0.053 | Reference category |  |
|  | 1-2 / month | 188 / 220 (85.5%) | 0.087 | 1.53 (0.94, 2.49) |  |
|  | 3-5 / month | 140 / 158 (88.6%) | 0.017 | 2.03 (1.13, 3.62) |  |
|  | >5 / month | 73 / 83 (88.0%) | 0.085 | 1.90 (0.92, 3.95) |  |
| Seizure frequency ^a^ | <1 / month | 192 / 242 (79.3%) | 0.009* | Reference category |  |
|  | >1 / month | 401 / 461 (87.0%) |  | 1.74 (1.15, 2.63) |  |
| Any antiepileptic treatment ^a^ | No | 39 / 55 (70.9%) | 0.009* | Reference category |  |
|  | Yes | 554 / 649 (85.4%) |  | 2.39 (1.29, 4.45) |  |
| Phenobarbital | No | 160 / 196 (81.6%) | 0.246 | Reference category |  |
|  | Yes | 433 / 508 (85.2%) |  | 1.30 (0.84, 2.01) |  |
| Potassium bromide | No | 427 / 511 (83.6%) | 0.422 | Reference category |  |
|  | Yes | 166 / 193 (86.0%) |  | 1.21 (0.76, 1.93) |  |
| Imepitoin | No | 557 / 661 (84.3%) | 0.925 | Reference category |  |
|  | Yes | 36 / 43 (83.7%) |  | 0.96 (0.42, 2.22) |  |
| Levetiracetam ^a^ | No | 214 / 269 (79.6%) | 0.008* | Reference category |  |
|  | Yes | 379 / 435 (87.1%) |  | 1.74 (1.16, 2.62) |  |
| Gabapentinoids | No | 530 / 630 (84.1%) | 0.821 | Reference category |  |
|  | Yes | 63 / 74 (85.1%) |  | 1.08 (0.55, 2.12) |  |
| Benzodiazepines ^a^ | No | 456 / 552 (82.6%) | 0.018* | Reference category |  |
|  | Yes | 137 / 152 (90.1%) |  | 1.92 (1.08, 3.42) |  |
| Zonisamide ^a^ | No | 510 / 614 (83.1%) | 0.016* | Reference category |  |
|  | Yes | 83 / 90 (92.2%) |  | 2.42 (1.09, 5.38) |  |
| Topiramate | No | 587 / 695 (84.5%) | 0.191 | Reference category |  |
|  | Yes | 6 / 9 (66.7%) |  | 0.37 (0.09, 1.49) |  |
| Heart conditions | No | 565 / 671 (84.2%) | 0.920 | Reference category |  |
|  | Yes | 28 / 33 (84.9%) |  | 1.05 (0.40, 2.78) |  |

^a^ included in the multivariable analysis based on p<0.15

^b^ turned into binary variable for inclusion in the multivariable model

^c^ in the case of multi-categorical variables, p-values located at the level of reference category and adjusted to the left side of the column indicate the result of the overall maximum likelihood G test while p-values adjusted to the right side of the column indicate results of comparison of the category at whose level they are located with the reference category

* significant at α=0.05

Table S10. Multivariable analysis of association between demographic and clinical characteristics of dogs and the presence of cardiorespiratory autonomic signs

| Variables | Regression coefficient (standard error) | Wald's statistic | p-value | Adjusted odds ratio  (95% confidence interval) |
| --- | --- | --- | --- | --- |
| Intercept | 0.20 (0.34) | - | - | - |
| General tonic-clonic seizures (GTCS) |  | | | |
| Not observed | Reference category | - | - | 1.00 |
| Observed | 0.97 (0.33) | 8.54 | 0.003* | 2.64 (1.38, 5.05) |
| Seizure frequency |  | | | |
| <1 episode / month | Reference category | - | - | 1.00 |
| ≥1 episode / month | 0.48 (0.22) | 4.85 | 0.028* | 1.62 (1.06, 2.49) |
| Antiepileptic treatment with levetiracetam |  | | | |
| Not used | Reference category | - | - | 1.00 |
| Used | 0.44 (0.22) | 4.19 | 0.041* | 1.56 (1.02, 2.38) |
| Labrador Retriever breed |  | | | |
| No | Reference category | - | - | 1.00 |
| Yes | 1.50 (0.74) | 4.15 | 0.042* | 4.48 (1.06, 19.0) |

Hosmer-Lemeshow χ^2^ test: p=0.946; Nagelkerke’s pseudo-R^2^ coefficient = 0.063; BIC = 616.7

* significant at α=0.05

Table S11. Univariable analysis of association between demographic and clinical characteristics of dogs and the presence of ocular autonomic signs. N indicates the number of dogs for which the information was available

| Dog characteristics | Category | No. (%) of dogs with ocular autonomic signs in the category | p-value ^c^ | Odds ratio (95% confidence interval) |
| --- | --- | --- | --- | --- |
| Demographic characteristics | | | | |
| Sex | Females | 192 / 252 (76.2%) | 0.356 | Reference category |
|  | Males | 358 / 452 (79.2%) |  | 1.19 (0.82, 1.72) |
| Neuter status | Entire | 141 / 174 (81.0%) | 0.279 | Reference category |
|  | Neutered | 409 / 530 (77.2%) |  | 0.79 (0.51, 1.22) |
| Body size | Miniature (<5 kg) | 29 / 36 (80.6%) | 0.873 | Reference category |
|  | Small (5-10 kg) | 78 / 106 (73.6%) | 0.393 | 0.67 (0.26, 1.71) |
|  | Medium (10-20 kg) | 137 / 172 (79.7%) | 0.902 | 0.94 (0.38, 2.34) |
|  | Large (20-40 kg) | 234 / 299 (78.3%) | 0.749 | 0.87 (0.36, 2.07) |
|  | Giant (>40 kg) | 34 / 44 (77.3%) | 0.720 | 0.82 (0.28, 2.43) |
|  | Unknown | 38 / 47 (80.9%) | 0.973 | - |
| Skull shape | Non-brachycephalic | 433 / 555 (78.0%) | 0.886 | Reference category |
|  | Brachycephalic | 79 / 102 (77.5%) | 0.899 | 0.97 (0.58, 1.61) |
|  | Unknown | 38 / 47 (80.9%) | 0.645 | - |
| Border collie | No | 511 / 654 (78.1%) | 0.982 | Reference category |
|  | Yes | 39 / 50 (78.0%) |  | 0.99 (0.50, 1.99) |
| Labrador Retriever | No | 519 / 661 (78.5%) | 0.337 | Reference category |
|  | Yes | 31 / 43 (72.1%) |  | 0.71 (0.35, 1.41) |
| Golden Retriever | No | 518 / 665 (77.9%) | 0.533 | Reference category |
|  | Yes | 32 / 39 (82.1%) |  | 1.30 (0.56, 3.00) |
| Age | <6 years | 336 / 428 (78.5%) | 0.836 | Reference category |
|  | 6–10 years | 158 / 202 (78.2%) | 0.983 | 0.98 (0.66, 1.48) |
|  | >10 years | 55 / 73 (75.3%) | 0.837 | 0.84 (0.47, 1.49) |
| Clinical characteristics | | | | |
| Age at which seizures started | <1 year | 115 / 142 (81.0%) | 0.599 | Reference category |
|  | 1–6 years | 389 / 501 (77.6%) | 0.815 | 0.82 (0.51, 1.30) |
|  | >6 years | 46 / 61 (75.4%) | 0.720 | 0.72 (0.35, 1.48) |
| Seizure type | Non-GTCS | 37 / 48 (77.1%) | 0.857 | Reference category |
|  | GTCS | 513 / 656 (78.2%) |  | 1.07 (0.53, 2.14) |
| Number of seizure episodes / month [N=703] ^b^ | <1 / month | 180 / 242 (74.4%) | 0.038* | Reference category |
|  | 1-2 / month | 177 / 220 (80.5%) | 0.121 | 1.42 (0.91, 2.20) |
|  | 3-5 / month | 120 / 158 (75.9%) | 0.723 | 1.09 (0.68, 1.73) |
|  | >5 / month | 73 / 83 (88.0%) | 0.012 | 2.51 (1.22, 5.17) |
| Seizure frequency ^a^ | <1 / month | 180 / 242 (74.4%) | 0.075 | Reference category |
|  | >1 / month | 370 / 461 (80.3%) |  | 1.40 (0.97, 2.02) |
| Seizure frequency ^a^ | <5 / month | 477 / 620 (76.9%) | 0.015* | Reference category |
|  | >5 / month | 73 / 83 (88.0%) |  | 2.19 (1.10, 4.35) |
| Any antiepileptic treatment ^a^ | No | 37 / 55 (67.3%) | 0.052 | Reference category |
|  | Yes | 513 / 649 (79.0%) |  | 1.84 (1.01, 3.32) |
| Phenobarbital ^a^ | No | 145 / 196 (74.0%) | 0.103 | Reference category |
|  | Yes | 405 / 508 (79.7%) |  | 1.38 (0.94, 2.03) |
| Potassium bromide | No | 401 / 511 (78.5%) | 0.717 | Reference category |
|  | Yes | 149 / 193 (77.2%) |  | 0.93 (0.62, 1.38) |
| Imepitoin | No | 520 / 661 (78.7%) | 0.187 | Reference category |
|  | Yes | 30 / 43 (69.8%) |  | 0.63 (0.32, 1.23) |
| Levetiracetam ^a^ | No | 200 / 269 (74.3%) | 0.058 | Reference category |
|  | Yes | 350 / 435 (80.5%) |  | 1.42 (0.99, 2.04) |
| Gabapentinoids | No | 491 / 630 (77.9%) | 0.722 | Reference category |
|  | Yes | 59 / 74 (79.7%) |  | 1.11 (0.61, 2.02) |
| Benzodiazepines ^a^ | No | 421 / 552 (76.3%) | 0.019* | Reference category |
|  | Yes | 129 / 152 (84.9%) |  | 1.75 (1.07, 2.83) |
| Zonisamide ^a^ | No | 472 / 614 (76.9%) | 0.027* | Reference category |
|  | Yes | 78 / 90 (86.7%) |  | 1.96 (1.04, 3.69) |
| Topiramate ^a^ | No | 545 / 695 (78.4%) | 0.130 | Reference category |
|  | Yes | 5 / 9 (55.6%) |  | 0.34 (0.09, 1.30) |
| Concomitant ocular conditions | No | 541 / 693 (78.1%) | 0.760 | Reference category |
|  | Yes | 9 / 11 (81.8%) |  | 1.26 (0.27, 5.91) |

^a^ included in the multivariable analysis based on p<0.15

^b^ turned into binary variable for inclusion in the multivariable model

^c^ in the case of multi-categorical variables, p-values located at the level of reference category and adjusted to the left side of the column indicate the result of the overall maximum likelihood G test while p-values adjusted to the right side of the column indicate results of comparison of the category at whose level they are located with the reference category

* significant at α=0.05

Table S12. Multivariable analysis of association between demographic and clinical characteristics of dogs and the presence of ocular autonomic signs

| Variables | Regression coefficient (standard error) | Wald's statistic | p-value | Adjusted odds ratio  (95% confidence interval) |
| --- | --- | --- | --- | --- |
| Intercept | 1.11 (0.10) | - | - | - |
| Antiepileptic treatment with benzodiazepines |  | | | |
| Not used | Reference category | - | - | 1.00 |
| Used | 0.52 (0.25) | 4.40 | 0.036* | 1.68 (1.04, 2.74) |
| Seizure frequency |  | | | |
| ≤5 episodes / month | Reference category | - | - | 1.00 |
| >5 episodes / month | 0.75 (0.35) | 4.53 | 0.033* | 2.11 (1.06, 4.21) |

Hosmer-Lemeshow χ^2^ test: p=0.596; Nagelkerke’s pseudo-R^2^ coefficient = 0.023; BIC = 745.7

* significant at α=0.05

Table S13. Univariable analysis of association between demographic and clinical characteristics of dogs and the presence of cutaneous autonomic signs. N indicates the number of dogs for which the information was available

| Dog characteristics | Category | No. (%) of dogs with cutaneous autonomic signs in the category | p-value ^c^ | Odds ratio (95% confidence interval) |
| --- | --- | --- | --- | --- |
| Demographic characteristics | | | | |
| Sex | Females | 132 / 252 (52.4%) | 0.989 | Reference category |
|  | Males | 237 / 452 (52.4%) |  | 1.00 (0.74, 1.36) |
| Neuter status | Entire | 88 / 174 (50.6%) | 0.576 | Reference category |
|  | Neutered | 281 / 530 (53.0%) |  | 1.10 (0.78, 1.55) |
| Body size ^b^ | Miniature (<5 kg) | 16 / 36 (44.4%) | 0.101 | Reference category |
|  | Small (5-10 kg) | 43 / 106 (40.6%) | 0.684 | 0.85 (0.40, 1.83) |
|  | Medium (10-20 kg) | 94 / 172 (54.7%) | 0.265 | 1.51 (0.73, 3.10) |
|  | Large (20-40 kg) | 166 / 299 (55.5%) | 0.209 | 1.56 (0.78, 3.13) |
|  | Giant (>40 kg) | 26 / 44 (59.1%) | 0.191 | 1.81 (0.74, 4.40) |
|  | Unknown | 24 / 47 (51.1%) | 0.550 | - |
| Body size ^a^ | Miniature or Small | 59 / 142 (41.6%) | 0.012* | Reference category |
|  | Medium, Large or Giant | 286 / 515 (55.5%) | 0.003 | 1.76 (1.21, 2.56) |
|  | Unknown | 24 / 47 (51.1%) | 0.256 | - |
| Skull shape | Non-brachycephalic | 286 / 555 (51.5%) | 0.492 | Reference category |
|  | Brachycephalic | 59 / 102 (57.8%) | 0.240 | 1.29 (0.84, 1.98) |
|  | Unknown | 24 / 47 (51.1%) | 0.951 | - |
| Border collie | No | 343 / 654 (52.4%) | 0.951 | Reference category |
|  | Yes | 26 / 50 (52.0%) |  | 0.98 (0.55, 1.75) |
| Labrador Retriever | No | 346 / 661 (52.3%) | 0.884 | Reference category |
|  | Yes | 23 / 43 (53.5%) |  | 1.05 (0.56, 1.94) |
| Golden Retriever | No | 348 / 665 (52.3%) | 0.854 | Reference category |
|  | Yes | 21 / 39 (53.8%) |  | 1.06 (0.56, 2.03) |
| Age ^b^ | <6 years | 229 / 428 (53.5%) | 0.304 | Reference category |
|  | 6–10 years | 107 / 202 (53.0%) | 0.900 | 0.98 (0.70, 1.37) |
|  | >10 years | 32 / 73 (43.8%) | 0.128 | 0.68 (0.41, 1.12) |
| Age ^a^ | ≤10 years | 336 / 630 (53.3%) | 0.124 | Reference category |
|  | >10 years | 32 / 73 (43.8%) |  | 0.68 (0.42, 1.11) |
| Clinical characteristics | | | | |
| Age at which seizures started | <1 year | 69 / 142 (48.6%) | 0.543 | Reference category |
|  | 1–6 years | 269 / 501 (53.7%) | 0.283 | 1.23 (0.84, 1.78) |
|  | >6 years | 31 / 61 (50.8%) | 0.771 | 1.09 (0.60, 1.99) |
| Seizure type | Non-GTCS | 26 / 48 (54.2%) | 0.801 | Reference category |
|  | GTCS | 343 / 656 (52.3%) |  | 0.93 (0.51, 1.67) |
| Number of seizure episodes / month [N=703] ^b^ | <1 / month | 115 / 242 (47.5%) | 0.151 | Reference category |
|  | 1-2 / month | 128 / 220 (58.2%) | 0.022 | 1.54 (1.06, 2.22) |
|  | 3-5 / month | 82 / 158 (51.9%) | 0.392 | 1.19 (0.80, 1.78) |
|  | >5 / month | 44 / 83 (53.0%) | 0.388 | 1.25 (0.76, 2.05) |
| Seizure frequency ^a^ | <1 / month | 115 / 242 (47.5%) | 0.056 | Reference category |
|  | >1 / month | 254 / 461 (55.1%) |  | 1.36 (0.99, 1.85) |
| Any antiepileptic treatment | No | 28 / 55 (50.9%) | 0.816 | Reference category |
|  | Yes | 341 / 649 (52.5%) |  | 1.07 (0.62, 1.85) |
| Phenobarbital | No | 98 / 196 (50.0%) | 0.426 | Reference category |
|  | Yes | 271 / 508 (53.3%) |  | 1.14 (0.82, 1.59) |
| Potassium bromide | No | 272 / 511 (53.2%) | 0.482 | Reference category |
|  | Yes | 97 / 193 (50.3%) |  | 0.89 (0.64, 1.24) |
| Imepitoin | No | 347 / 661 (52.5%) | 0.865 | Reference category |
|  | Yes | 22 / 43 (51.2%) |  | 0.95 (0.51, 1.76) |
| Levetiracetam | No | 143 / 269 (53.2%) | 0.756 | Reference category |
|  | Yes | 226 / 435 (52.0%) |  | 0.95 (0.70, 1.29) |
| Gabapentinoids | No | 326 / 630 (51.7%) | 0.299 | Reference category |
|  | Yes | 43 / 74 (58.1%) |  | 1.29 (0.79, 2.11) |
| Benzodiazepines | No | 286 / 552 (51.8%) | 0.541 | Reference category |
|  | Yes | 83 / 152 (54.6%) |  | 1.12 (0.78, 1.60) |
| Zonisamide | No | 322 / 614 (52.4%) | 0.969 | Reference category |
|  | Yes | 47 / 90 (52.2%) |  | 0.99 (0.64, 1.54) |
| Topiramate | No | 366 / 695 (52.7%) | 0.246 | Reference category |
|  | Yes | 3 / 9 (33.3%) |  | 0.45 (0.11, 1.81) |
| Concomitant skin and ear conditions ^a^ | No | 335 / 652 (51.4%) | 0.050* | Reference category |
|  | Yes | 34 / 52 (65.4%) |  | 1.79 (0.99, 3.23) |

^a^ included in the multivariable analysis based on p<0.15

^b^ turned into binary variable for inclusion in the multivariable model

^c^ in the case of multi-categorical variables, p-values located at the level of reference category and adjusted to the left side of the column indicate the result of the overall maximum likelihood G test while p-values adjusted to the right side of the column indicate results of comparison of the category at whose level they are located with the reference category

* significant at α=0.05

Table S14. Multivariable analysis of association between demographic and clinical characteristics of dogs and the presence of cutaneous autonomic signs

| Variables | Regression coefficient (standard error) | Wald's statistic | p-value | Adjusted odds ratio  (95% confidence interval) |
| --- | --- | --- | --- | --- |
| Intercept | -0.43 (0.18) | - | - | - |
| Skin and Ear problems |  | | | |
| Not reported | Reference category | - | - | 1.00 |
| Reported | 0.66 (0.31) | 4.59 | 0.032* | 1.93 (1.06, 3.51) |
| Body size |  | | | |
| Miniature/small | Reference category | - | - | 1.00 |
| Medium/Large/Giant | 0.61 (0.19) | 9.98 | 0.002* | 1.85 (1.26, 2.71) |
| Unknown | 0.45 (0.34) | 1.71 | 0.191 | 1.57 (0.80, 3.07) |

Hosmer-Lemeshow χ^2^ test: p=0.786; Nagelkerke’s pseudo-R^2^ coefficient = 0.026; BIC = 983.7

* significant at α=0.05
